# Studying independent *Kcna6* knock-out mice reveals toxicity of exogenous LacZ to central nociceptor terminals and differential effects of Kv1.6 on acute and neuropathic pain sensation

**DOI:** 10.1523/JNEUROSCI.0187-21.2021

**Published:** 2021-09-20

**Authors:** LJ Peck, R Patel, P Diaz, YM Wintle, AH Dickenson, AJ Todd, M Calvo, DLH Bennett

**Affiliations:** 1 Nuffield Department of Clinical Neurosciences, University of Oxford, Oxford OX3 9DU, UK; 2 Department of Neuroscience, Physiology and Pharmacology, University College London, London WC1E 6BT, UK; 3 Departamento de Fisiologia, Facultad de Ciencias Biologicas, Pontificia Universidad Catolica de Chile and Millennium Nucleus for the Study of Pain (MiNuSPain), Santiago 8330025, Chile; 4 Institute of Neuroscience and Psychology, University of Glasgow, Glasgow G12 8QQ, UK

## Abstract

The potassium channel Kv1.6 has recently been implicated as a major modulatory channel subunit expressed in primary nociceptors. Furthermore, its expression at juxtaparanodes (JXP) of myelinated primary afferents is induced following traumatic nerve injury as part of an endogenous mechanism to reduce hyperexcitability and pain-related hypersensitivity. In this study we compared two mouse models of constitutive Kv1.6 knock-out achieved by different methods: traditional gene trap via homologous recombination, and CRISPR-mediated excision. Both Kv1.6 knock-out mouse lines exhibited an unexpected reduction in sensitivity to noxious heat stimuli, to differing extents: the Kv1.6 mice produced via gene trap had a far more significant hyposensitivity. These mice (*Kcna6^lacZ^*) expressed the bacterial reporter enzyme LacZ in place of Kv1.6 as a result of the gene trap mechanism and we found that their central primary afferent presynaptic terminals developed a striking neurodegenerative phenotype involving accumulation of lipid species, development of ‘meganeurites’ and impaired transmission to dorsal horn wide dynamic range (WDR) neurons. The anatomical defects were absent in CRISPR-mediated Kv1.6 knock-out mice (*Kcna6* ^-/-^) but were present in a third mouse model expressing exogenous LacZ in nociceptors under the control of a Nav1.8-promoted Cre recombinase. LacZ reporter enzymes are thus intrinsically neurotoxic to sensory neurons and may induce pathological defects in transgenic mice, which has confounding implications for the interpretation of gene knock-outs using *lacZ*. Nonetheless, in *Kcna6* ^-/-^ mice not affected by LacZ, we demonstrated a significant role for Kv1.6 regulating acute noxious thermal sensitivity, and both mechanical and thermal pain-related hypersensitivity after nerve injury.

## Introduction

The *Shaker*-like Kv1 potassium channel family comprises 8 alpha subunits (Kv1.1-1.8), each considered a mammalian homolog of the *Shaker* channel that produced hyperexcitable sensorimotor phenotypes in *Drosophila* when mutated. These channels have also been shown to be key regulators of neuronal excitability in mammals (Browne et al., 1994; Smart et al., 1998; Robbins and Tempel, 2012). The most comprehensive recent reports indicate that Kv1.1, Kv1.2, Kv1.4 and Kv1.6 are expressed in mouse sensory neurons (Zeisel et al., 2018; Zheng et al., 2019). Although mouse knock-out studies have shown that Kv1.1 and Kv1.2 regulate dorsal root ganglion (DRG) neuron excitability, there have been few studies of the role of Kv1.6 (encoded by *Kcna6*) in DRG neurons and in sensory coding.

Recent single-cell transcriptomic approaches detected expression of *Kcna6* mRNA throughout the mouse nervous system (Usoskin et al., 2015; Häring et al., 2018; Zeisel et al., 2018) and, within sensory ganglia, *Kcna6* is reported to be the second most abundant potassium channel subunit in nociceptive populations (Zheng et al., 2019). Blockade of Kv1.1/1.2/1.6-containing channels by α-dendrotoxin (α-DTx) in Mrgprd-expressing neurons increased the repetitive firing capability of these nociceptive neurons *in vitro* (Zheng et al., 2019).

Kv1.6 is upregulated in rodent and human myelinated primary somatosensory neurons after peripheral nerve injury (Calvo et al., 2016). Under these conditions, Kv1.6 subunits emerge at neuronal sites flanking the node of Ranvier and replace native subunits Kv1.1 and Kv1.2, which are known to be downregulated transcriptionally and at the protein level shortly after peripheral nerve injury (Ishikawa et al., 1999; Zhao et al., 2013; Calvo et al., 2016; Hong et al., 2016; González et al., 2017). The timing of Kv1.6 appearance at the JXP and paranode corresponds with both a reduction of ectopic electrical activity and improved withdrawal thresholds for reflexes evoked mechanically by Von Frey hairs (Calvo et al., 2016). However, both of these recovery effects were reversed by pharmacological blockade of Kv1.6-containing channels with local application of α-DTx (Calvo et al., 2016). It is therefore suggested that Kv1.6 subunits are involved in a compensatory response to peripheral nerve injury.

These data suggest that Kv1.6-containing channels provide ‘brake’-like counter-currents that oppose neural excitation in sensory neurons, as described for other Kv1 subunits (Madrid et al., 2009; Hao et al., 2013; Zhao et al., 2013; González et al., 2017; Dawes et al., 2018). In this study, we have sought to detail the expression of *Kcna6* in the mouse sensory neuraxis, with a predominant focus on primary afferent neurons of the dorsal root ganglia (DRG). In addition to this, through a global gene knock-out strategy, we have studied sensorimotor behaviour in two *Kcna6*-null mouse strains (termed *Kcna6^lacZ/lacZ^* and *Kcna6* ^-/-^) to identify a role for Kv1.6-containing channels in pain and/or somatosensation in the contexts of acute and neuropathic pain. Surprisingly, results were not consistent between *Kcna6* knock-out strains and we provide anatomical, electrophysiological and behavioural evidence that the presence or absence of exogenous *lacZ* reporter cassettes accounts for the phenotypic differences between these two mouse strains. Furthermore, we show that expression of *lacZ* alone on an otherwise phenotypically normal background is sufficient to cause deleterious effects to nociceptive presynaptic terminals in the dorsal horn. Notwithstanding these unexpected consequences of genetic manipulation, the findings of this study support a role for *Kcna6* in acute noxious thermal sensation and in contributing to recovery of normal sensory function in a neuropathic pain model.

## Methods and Materials

### Animals

Behavioural experiments involving the use of uninjured mice were performed in compliance with the Animals (Scientific Procedures) Act 1986, under UK Home Office-issued project licenses 30/3015 and P1DBEBAB9 held by Prof David Bennett at the University of Oxford. Behavioural experiments involving the chronic constriction injury (CCI) model of neuropathy were performed at Pontificia Universidad Católica de Chile and approved by the Institutional Ethics Committee (protocol ID 150714013). *In vivo* electrophysiological experiments were performed at University College London under UK Home Office project licence PEB669065 held by Prof Anthony Dickenson. Male and female mice were used throughout.

*Kcna6^lacZ^ mice*: *Kcna6* ^tm1Lex^/Mmucd mice were purchased from MMRRC (RRID: MMRRC_011729-UCD) on a mixed C57BL/6J;129S5 background, with heterozygote or wild-type littermates used as controls in all experiments. The *Kcna6* ^tm1Lex^ allele was produced by Lexicon Genetics, Inc. (now Lexicon Pharmaceuticals, Inc.) by homologous recombination of a gene trap cassette, containing *lacZ*/*neo* selection genes, with the coding sequence of *Kcna6* exon 1, depicted in (Fig. 3A). *Kcna6^lacZ/lacZ^*; *Advillin*-EGFP mice: In order to label primary afferent terminals within the spinal cord, the *Kcna6^lacZ^* strain was interbred with a mouse strain expressing a BAC transgene encoding EGFP under the *Advillin* promoter – Tg(Avil-EGFP)QD84Gsat/Mmucd (RRID: MMRRC_034769-UCD). *Kcna6*^-/-^ mice: *Kcna6* ^em1(IMPC)J^/Mmjax mice were purchased from MMRRC (RRID: MMRRC_042391-JAX) and maintained on a C57BL/6NJ background, with heterozygote or wild-type littermates used as controls in all experiments. The nature of the mutation is Cas9-mediated excision of the sole coding region, leaving the initial 7 residues intact and resulting in frame shift and premature STOP codon. Nav1.8^Cre/+^; ROSA26^*lacZ*/+ mice:^ The *Gt(ROSA)26Sor^tm1Sor^/J* mouse is a conditional-ready strain carrying a *loxP–neo–polyA–loxP–lacZ* cassette in the ubiquitously expressed ROSA26 locus (Soriano, 1999). Thus, there is ubiquitous expression of neomycin phosphotransferase (*neo*), followed by a *polyA* tail, which ordinarily prevents read-through transcription of *lacZ*. The *neo–polyA* sequence is flanked by *loxP* sites, so Cre-mediated recombination excises this region and results in expression only of *lacZ*. Homozygous conditional-ready *Gt(ROSA)26Sor^tm1Sor^/J* mice were gifted kindly by Prof Shankar Srinivas (DPAG, University of Oxford) to serve as founders of the Nav1.8^Cre/+^; ROSA26^*lacZ*/+^ colony by crossing with in-house heterozygous Nav1.8 Cre mice (Nassar et al., 2004; Stirling et al., 2005) gifted by Prof John Wood, University College London. This produced compound heterozygotes expressing one *lacZ* allele at the ROSA26 locus in Nav1.8-positive neurons. Nav1.8 Cre-negative littermates did not conditionally express LacZ, but constitutively expressed a neomycin phosphotransferase cassette at the ROSA26 locus.

### Tissue preparation for immunohistochemistry and *in situ* hybridisation

To extract tissue for cryo-sectioning, animals were culled by overdose of pentobarbital delivered via intraperitoneal injection, followed by transcardial perfusion with 15ml sterile 0.9% w/v saline, then 20ml 4% paraformaldehyde (PFA) in 0.1M phosphate buffer (PB). Dissected tissues were post-fixed in 4% PFA for varied duration depending on tissue, as determined previously in our laboratory (Dawes et al., 2018): L4 DRG – 2hrs (room temperature, RT), spinal cord – 24hrs (4°C), glabrous skin – 0.5hrs (RT). For analysis of epidermal nerve fibres, skin was dissected from the glabrous region of the hind paw proximal to the most proximal touch dome, as depicted in (Fig. 6A). After fixation, tissue was transferred to 30% sucrose in 0.1M PB at 4°C for at least 24 hours. Subsequently, tissue was embedded in OCT medium (TissueTek), rapidly frozen with liquid nitrogen, and stored at -80°C prior to cryo-sectioning. Sections were slide-mounted on Thermo Scientific™ SuperFrost Plus™ slides – with sections cut using a Leica cryostat, at tissue-dependent thickness: DRG – 10μm; spinal cord – 20μm, skin – 14μm.

### *In situ* hybridisation

Expression of *Kcna6* mRNA was investigated using a chromogenic *in situ* hybridisation assay (RNAScope 2.5-RED, Advanced Cell Diagnostics). In brief, slides were removed from -80°C storage and allowed to equilibrate to room temperature prior to removal of OCT by submersion in DNAase/RNAase-free phosphate-buffered saline (PBS). Slides were pre-treated with a 10-minute application of H_2_O_2_ at room temperature, washed with double deionised Milli-Q water, submerged in 100% EtOH and allowed to dry (20 mins) before a 10-minute application of protease plus in a humidified chamber at 40°C, followed by a further Milli-Q wash. The *Kcna6*-specific target detection probe (Cat No. 462821) was warmed in a 40°C water bath prior to a 2-hour incubation on slides at 40°C, and the amplification steps and fast red reaction were performed according to the manufacturer’s instructions. Following development of the red signal, slides were counter-stained using the protocol described below.

### Immunohistochemistry

Before antibody application, slides were washed and sections concurrently permeabilised in PBS+0.3% Triton X-100. Primary antibodies were diluted in PBS containing 0.3% Triton X-100 and 5% goat or donkey blocking serum and incubated on sections overnight in a humidified chamber. The next day, unbound primary antibodies were washed off with PBS, and secondary antibodies were applied to sections for 2-3 hours, followed by further washing in PBS and, in some instances where a biotinylated secondary antibody was used, a tertiary antibody incubation for up to 2 hours. Finally, slides were mounted with glass coverslips and Vectashield mounting medium for fluorescence, and air-sealed with nail polish. For ganglioside antibody staining, slides were washed for 3mins in PBS + 0.1% Tween to avoid excess permeabilisation; other steps in this protocol were the same as previously described for immunohistochemistry. Exact antibodies and dilutions used are detailed in the Appendix.

### Image acquisition

Fluorescence imaging was performed with a Zeiss LSM700 confocal microscope. For quantification, images were generally acquired using a 20x, 40x or 63x objective. Maximum intensity projections were produced from z-stacks encompassing the entire section depth. For *in situ* hybridisation experiments, image acquisition settings were kept the same for all quantified images in order to make signal comparable across sections.

### RNA extraction and cDNA synthesis

Mice were culled by rising CO_2_ concentration in a sealed chamber. Brains were rapidly dissected, immediately frozen in liquid nitrogen, and stored at -80°C. Tissue was homogenised in TriPure (Roche) and then treated with chloroform prior to column purification using a High Pure RNA isolation kit (Roche). RNA was eluted in RNAase-free water. Synthesis of cDNA was achieved using Transcriptor reverse transcriptase (Roche) with random hexamers (Invitrogen) and dNTPs (Roche).

### RT-qPCR

Quantitative analysis of mRNA expression was carried out on a LightCycler 480 II system (Roche), using SYBR green fluorescence for detection. Primers were designed using Primer-BLAST (https://www.ncbi.nlm.nih.gov/tools/primer-blast/) and were assessed for specificity and efficiency by melting curve analysis, and by serial dilution. Primers (0.5μM) and cDNA (5ng) were mixed with LightCycler 480 SYBR Green master mix (Roche) in a 1:1 ratio in 384-well plates and run on a 45-cycle protocol. All experimental samples were run in triplicate. Quantitation of expression was performed using the delta delta CT method (Livak and Schmittgen, 2001), and Kv1 gene products were first normalised to ‘housekeeping’ genes (*Gapdh* and *Hprt1*) and then normalised to average wild-type expression per gene. Primer sequences are detailed in the Appendix.

### Behavioural assays

Mice were housed in individually ventilated cages with no more than 6 or no fewer than 2 mice per cage, and fed *ad libitum*. The light-dark cycle period was 12 hours, and behavioural experiments were performed during the light cycle. Multiple different behavioural assays were performed each day, but the time of day for each behavioural assay was kept constant. Prior to all experiments, animals were habituated to equipment so that the experimental environment was not novel during data collection. Unless otherwise stated, baseline results were averaged from 3 baseline recordings. Data from left and right hind limb paws were combined and averaged for Hargreaves, Von Frey, pin-prick and dry ice results. The experimenter was blinded to genotype at all times during behavioural testing and analysis. Details of individual assays are as follows:

a) *Open field*
  Mice were placed in the corner of a 50x30cm wooden box with a 5x3 grid marked on the base and allowed to move freely for 3 minutes. During this time, the number of grid lines crossed and hind limb rearing episodes were recorded.
b) *Rota-rod*
  Mice were required to climb/walk against the direction of the platform (Ugo Basile) rotating at 28rpm beneath them. The designated end point of this experiment was when the animal falls off the equipment, or at the 90 second mark, or when they cling to and rotate with the platform for 2 full revolutions cumulatively throughout the 90 second period.
c) *Beam test*
  Mice were filmed from behind walking between two points along a thin cylindrical beam (approximately 1.5cm in diameter). The video footage was used to calculate the percentage of ‘incorrect’ hind limb steps (that is, where hind paws either slip or hop on the beam) from the total number of steps.
d) *Hot plate*
  Animals were placed on the hot plate (Ugo Basile) set to either 50˚C or 53˚C, at which point a timer was started by depression of a foot switch. The time was recorded, and the animal removed, when the animal displayed visible pain behaviour (hind paw licking, biting, shaking, or jumping) or after a maximum of 20 seconds at maximal temperature of 53°C to avoid tissue injury.
e) *Von Frey*
  Mice were housed individually in plexiglass enclosures on a metallic grille platform. After an acclimation period of between 30 minutes to 1 hour, 50% mechanical withdrawal thresholds were derived using the Dixon up-and-down method (Dixon, 1965). Baseline measurements were averaged from left and right paw.
f) *Hargreaves*
  Mice were housed individually in plexiglass enclosures on a glass platform. An infrared heat source (Ugo Basile) was placed under the animal, targeted through the glass at the hind paw. Switching on the heat source begins a timer, which turns off once the animal responds to the stimulus by relocating its paw. Otherwise, the test was stopped at a maximum of 20 seconds.
g) *Pin-prick*
  Animals were temporally housed individually in plexiglass chambers on a grille used for Von Frey testing. A sharp pin was fixed to the filament of a 2.0g Von Frey hair and used to provide noxious punctate stimulation to left and right hind-paws. Withdrawal reflexes were filmed from below using an iPhone8 in slo-mo mode (720p, 240fps) such that still frames are ~4ms apart. The withdrawal latency was derived post-hoc by scrolling through frames from the time of contact with the paw to withdrawal. Two or occasionally three stimuli were applied to each paw on three separate baseline testing days, from which a mean baseline latency was calculated.
h) *Dry ice (cold plantar assay)*
  This assay has been described in detail previously (Brenner et al., 2012). Briefly, mice were housed individually in plexiglass chambers on a 5mm borosilicate glass surface (UQG Optics Ltd.). Crushed dry ice pellets were compacted into a syringe with the tapered end cut off, such that the dry ice could be applied to the glass evenly beneath the hind-paw. A stopwatch was used to time the withdrawal latency.

### Neuropathic model – chronic constriction injury (CCI)

Chronic constriction injury was performed as described previously (Bennett and Xie, 1988). Mice were anesthetized using isoflurane inhalation. The left sciatic nerve was exposed at the high-thigh level. Proximal to its trifurcation, 3 ligatures with 6-0 silk suture were tied loosely around the nerve at intervals of around 2 mm. The wound was closed with three external stitches and disinfected. All behavioural measurements were taken in awake and unrestrained mice of both sexes (2-4 months old). Two sessions of habituation (40-50 min each) to the testing area were conducted in two consecutive days followed by two baseline measurements of hind paw withdrawal threshold in another two consecutive days. Groups were matched by sex and age. Mechanical and thermal sensitivity was assessed using Von Frey hairs and the Hargreaves apparatus as previously described in this manuscript. Thermal place preference was additionally used as a parameter to assess thermal hypersensitivity. Mice were placed at the centre of an apparatus that uses temperature adjustable plates. The reference plate was adjusted to set a neutral temperature at 25°C, whereas the other plate, was adjusted to the specific noxious temperature of interest (50°C or 53°C). The mouse was then allowed to move freely from plate to plate for 2 min. Movements were recorded by a camera mounted directly above the enclosed space. Data are expressed as the percentage of the time spent on the test plate.

### Intra-epidermal nerve fibre density

Following immunostaining, 3 sections each from 4 animals per *Kcna6^lacZ^* genotype were analysed on a Zeiss LSM700 confocal microscope. Using DAPI to mark the dermis/epidermis boundary, the number of Pgp9.5 immunoreactive fibres that could be visualised crossing from dermis to epidermis was counted through the viewfinder with a 63x oil immersion lens. A post hoc overview image was taken, and the length of epidermis analysed was measured in ZEN Black software; across all sections this ranged between 2.1-3.3mm.

### DRG subpopulation counts and Atf3 expression

For subpopulation analysis, at least 2 whole DRG sections were imaged and analysed per animal. DRG ‘profiles’ were defined as being Neurotrace-positive and nucleated. Profiles were designated positive or negative for histochemical markers by eye on images of whole DRG sections taken with identical acquisition settings, and their abundance was calculated as a percentage of Neurotrace-positive profiles (fluorescent Nissl stain). For Atf3 analysis, at least 4 randomly chosen fields of view were analysed from DRGs dissected from 4 *Kcna6^lacZ/lacZ^* animals and 4 wild-type littermates.

### Transmission electron microscopy

Adult mice received an intraperitoneal overdose of pentobarbital and were then transcardially perfused with 0.9% saline followed by a 1% PFA, 1% glutaraldehyde fixative in 0.1M phosphate buffer (PB). Spinal cords were then removed and post-fixed in the same solution for 24 hours and transferred to 0.1M PB before sectioning. 60μm sections were prepared using a vibrating-blate microtome, post-fixed with osmium tetroxide OsO_4_, dehydrated and flat-embedded in epoxy resin. At this stage, some images were acquired using light microscopy. Subsequently, ultrathin sections were cut using a Diatome diamond knife and Leica Ultracut S ultramicrotome. Images were acquired at 120kV on a Philips CM100 transmission electron microscope with a Gatan OneView CMOS camera. The appearance of identifiable glomerular structures was interpreted qualitatively.

### *In vivo* dorsal horn recordings

Both male and female *Kcna6* ^+/+^, *Kcna6^lacZ^* ^/+^, and *Kcna6^lacZ/lacZ^* littermate mice were used; the mean ages of animals ± standard deviation were 17.3±3.3 (*Kcna6* ^+/+^), 14.7±1.1 (*Kcna6^lacZ^* ^/+^), and 17.1±2.5 (*Kcna6^lacZ/lacZ^*) weeks. Mice were initially anaesthetised with 3.5% v/v isoflurane delivered in 3:2 ratio of nitrous oxide and oxygen. Once areflexic, mice were secured in a stereotaxic frame and subsequently maintained on 1.5% v/v isoflurane for the remainder of the experiment (approximately 4 hours in duration). Core body temperature was maintained with the use of a homeothermic blanket and respiratory rate was visually monitored throughout. A laminectomy was performed to expose the L3-L5 segments of the spinal cord; mineral oil was then applied to prevent dehydration. Extracellular recordings were made from deep dorsal horn wide dynamic range (WDR) neurons with receptive fields on the glabrous skin of the toes using 0.127mm 2MΩ parylene-coated tungsten electrodes (A-M Systems, Sequim, WA, USA). Searching involved light tapping of the hind paw whilst manually moving the electrode. All recordings were made at depths delineating the deep dorsal horn laminae (Watson et al., 2009), and were classified as wide dynamic range (WDR) on the basis of neuronal sensitivity to dynamic brushing (i.e. gentle stroking with a squirrel-hair brush), and noxious punctate mechanical (15g) and heat (48°C) stimulation of the receptive field.

The receptive field was then stimulated using a wider range of natural stimuli (brush, Von Frey filaments – 0.4, 1, 4, 8 and 15g, and heat – 32, 42, 45 and 48°C) applied over a period of 10s per stimulus and the evoked response quantified. The heat stimulus was applied with a constant water jet onto the centre of the receptive field. Ethyl chloride (50μl) was applied to the receptive field, described previously as an evaporative noxious cooling stimulus (Leith et al., 2010). Evoked responses to room temperature water (25°C) were subtracted from ethyl chloride-evoked responses to control for any concomitant mechanical stimulation during application. Natural stimuli were applied starting with the lowest intensity stimulus with approximately 40s between stimuli in the following order: brush, Von Frey, cold, heat, electrical. Receptive fields were determined using a 15g Von Frey. An area was considered part of the receptive field if a response of >30 action potentials over 5s was obtained. A rest period of 30s between applications was used to avoid sensitisation. Receptive field sizes are expressed as a percentage area of a standardised paw measured using ImageJ (NIH, Bethesda, MD). Electrical stimulation of WDR neurons was delivered transcutaneously via needles inserted into the receptive field. A train of 16 electrical stimuli (2ms pulses, 0.5Hz) was applied at three times the threshold current for C-fibre activation. Responses evoked by A-(0–50ms) and C-fibres (50–250ms) were separated and quantified on the basis of latency. Neuronal responses occurring after the C-fibre latency band were classed as post-discharge (PD). The input (I) and the wind-up (WU) were calculated as: [Input = action potentials evoked by first pulse × total number of pulses] and [Wind up = total action potentials after 16 train stimulus – Input].

The signal was amplified (x6000), bandpass filtered (low/high frequency cut-off 0.5/2kHz) and digitised at rate of 20kHz. Data were captured and analysed by a Cambridge Electronic Design 1401 interface coupled to a computer with Spike2 software (CED, Cambridge, UK) with post-stimulus time histogram and rate functions. In some cases where a single unit recording could not be obtained, spike sorting was performed post hoc with Spike2 using fast Fourier transform followed by 3-dimensional principal component analysis of waveform features for multi-unit discrimination (electrical stimulation was not performed for these neurons). One to three neurons were characterised per mouse. In total, 19 neurons were characterised from 14 wild-type mice (7 male, 7 female), 21 neurons from 16 *Kcna6^lacZ^* ^/+^ mice (10 male, 6 female), and 19 neurons from 15 *Kcna6^lacZ/lacZ^* mice (6 male, 9 female). Of these neurons, 15 per genotype were successfully stimulated transcutaneously so that electrical properties could be interrogated. In vivo electrophysiological procedures were non-recovery; at the end of experiments mice were terminally anaesthetised with isoflurane.

### Experimental design and statistical analyses

GraphPad Prism software was used for all statistical calculations. In general, unless otherwise stated, the unit of statistical analysis was the individual animal and significance is reported as multiplicity-adjusted p-values. *N* values and details of mouse sex are included in figure legends as appropriate.

**Figure 1:**
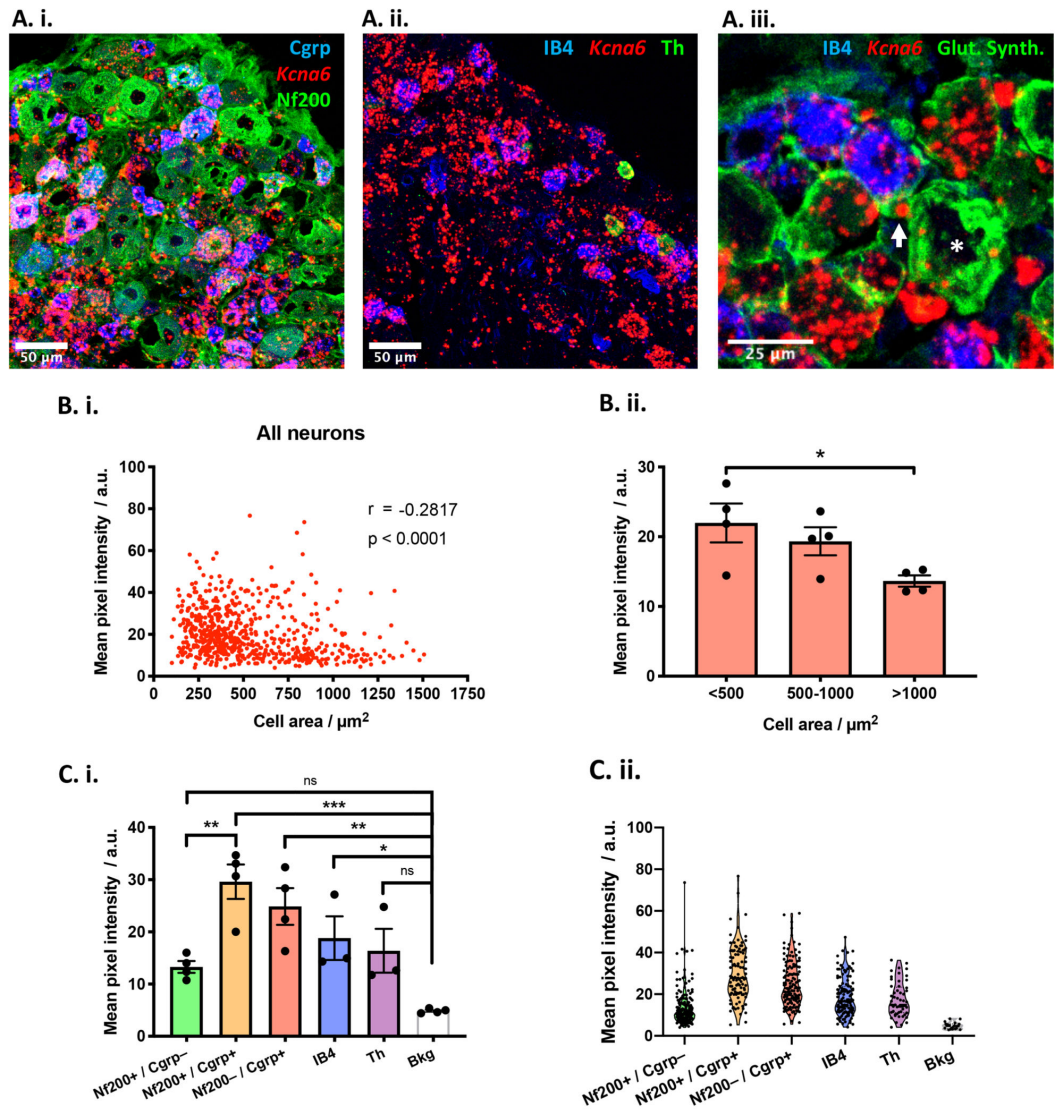
*Kcna6* mRNA is expressed in DRG neurons and satellite glia **(A)** Combined fluorescent in situ hybridisation and immunohistochemistry reveals *Kcna6* mRNA expression in the lumbar (L4) dorsal root ganglia. *Kcna6* in situ signal intensity was analysed in L4 DRGs immunostained for neuronal markers: Nf200 and Cgrp **(A.i.)**; IB4 and Th **(A.ii.)**; IB4 and satellite glial marker glutamine synthetase **(A.iii.)**. *Kcna6* is expressed in the Cgrp+ and IB4-binding neuronal sub-populations and at a lower level in the Th population. Some expression in satellite glia ensheathing an IB4-labelled neuron (arrow) and a larger, IB4-negative neuron (asterisk) are indicated. Scale bars = 50μm **(A.i-ii.)**, 25μm **(A.iii.)**. **(B.i.)** *Kcna6* expression across all DRG neurons analysed from 4 animals is negatively correlated with cell size (non-parametric two-tailed Spearman’s correlation, r = -0.2817, p<0.0001). **(B.ii.)** Small (<500μm2) diameter DRG neurons had higher expression of Kcna6 mRNA compared to large (>1000μm2) diameter DRG neurons. (n = 4 WT animals per category) * = p<0.05 <500 vs. >1000 (small vs. large) (Ordinary One-Way ANOVA with Tukey’s post-hoc multiple comparisons test). **(C.i.)** Differentiating DRG populations by histochemical markers reveals generally very low *Kcna6* expression in myelinated (Nf200+) neurons, but highest expression in myelinated peptidergic (Nf200+/Cgrp+) neurons. On per-animal analysis, expression is significantly above background (Bkg) in all nociceptive populations. Data points are mean values from individual replicates, plotted with overall WT mean ± SEM. n = 4 or 3 WT animals per category, */**/*** = p<0.05/0.01/0.001 (One-way ANOVA with Tukey’s post hoc multiple comparisons test). Bkg = background signal from attached nerve root. **(C.ii.)** Data points represent individual neurons, combined from all DRG sections from all animals.

*In situ hybridisation* – Expression of *Kcna6* mRNA was quantified by calculating the mean red channel pixel intensity (converted from 0-255 8-bit colour value to a percentage scale) in regions of interest (ROIs) drawn in imageJ software around cells with identifiable nuclei, thus expression is normalised to cell size (sum of pixel intensities ÷ number of pixels). ‘Background’ was from ROIs containing nerve root. Mean *Kcna6* expression per animal was assessed for normality using the Shapiro-Wilk test and compared between categories by Ordinary One-Way ANOVA with Tukey’s post-hoc multiple comparisons test (Fig. 1B.ii., C.i.).

*RT-qPCR* – Comparison of Kv1 subunit mRNA expression between *Kcna6^lacZ^* genotypes was performed using Ordinary One-Way ANOVA independently for each gene or primer, with Tukey’s post-hoc multiple comparisons test.

*Behaviour* – data are presented as group mean (grouped by genotype and/or time point) ± standard error of the mean (SEM) unless otherwise stated and include data points from individual animals. Data were first assessed for normality using the Shapiro-Wilk test. Baseline sensorimotor performance was compared between genotypes by One-Way ANOVA. We used Dunnett’s post hoc multiple comparisons test to identify differences relative to wild-type controls, or Tukey’s post hoc test when reporting differences with all possible comparisons between genotypes. For CCI studies, a Two-Way Repeated Measures ANOVA was used with Šidák multiple comparisons test to compare genotypes at different time points. The unit of statistical analysis was the animal, and significance was reported as multiplicity-adjusted p-values.

*IENFD* – samples were assessed for normality by Shapiro-Wilk test and means compared with a two-tailed, unpaired Student’s t-test.

*DRG populations* – samples were assessed for normality by Shapiro-Wilk test, within each subpopulation. Multiple t-tests were performed to compare wild-type and *Kcna6^lacZ/lacZ^* values for each subpopulation, corrected for with a false discovery rate of 5% using the recommended Benjamini, Krieger and Yekutieli method in GraphPad Prism.

*In vivo electrophysiology* – in all experiments, the neuron was considered to be the experimental unit of replication. Minimum group sizes were determined by a priori calculations using the following assumptions (α 0.05, 1-β 0.8, ε 1, effect size range d = 0.5 to 0.8). Effect sizes were based on historical data sets. Mechanical and heat responses were analysed by Two-Way Repeated Measures ANOVA with Dunnett’s post-hoc test to compare to wild-type results. Brush, cold, fibre threshold, electrical results, and receptive field were assessed for normality using the Shapiro-Wilk test. If they passed, each characteristic was assessed by One-Way ANOVA with Dunnett’s or Tukey’s post-hoc test to make multiple comparisons versus wild-types or between all genotypes, respectively. For characteristics which failed the normality test, a Kruskal-Wallis test was performed instead with Dunn’s post-hoc multiple comparisons test. Multiplicity-adjusted p-values are reported in each case. Kinetics of wind-up were assessed by non-linear regression, fitting a one-phase association curve to each data set and comparing the rate constant and plateau between each genotype.

## Results

### Expression of Kcna6 in the mouse DRG

**Figure 2:**
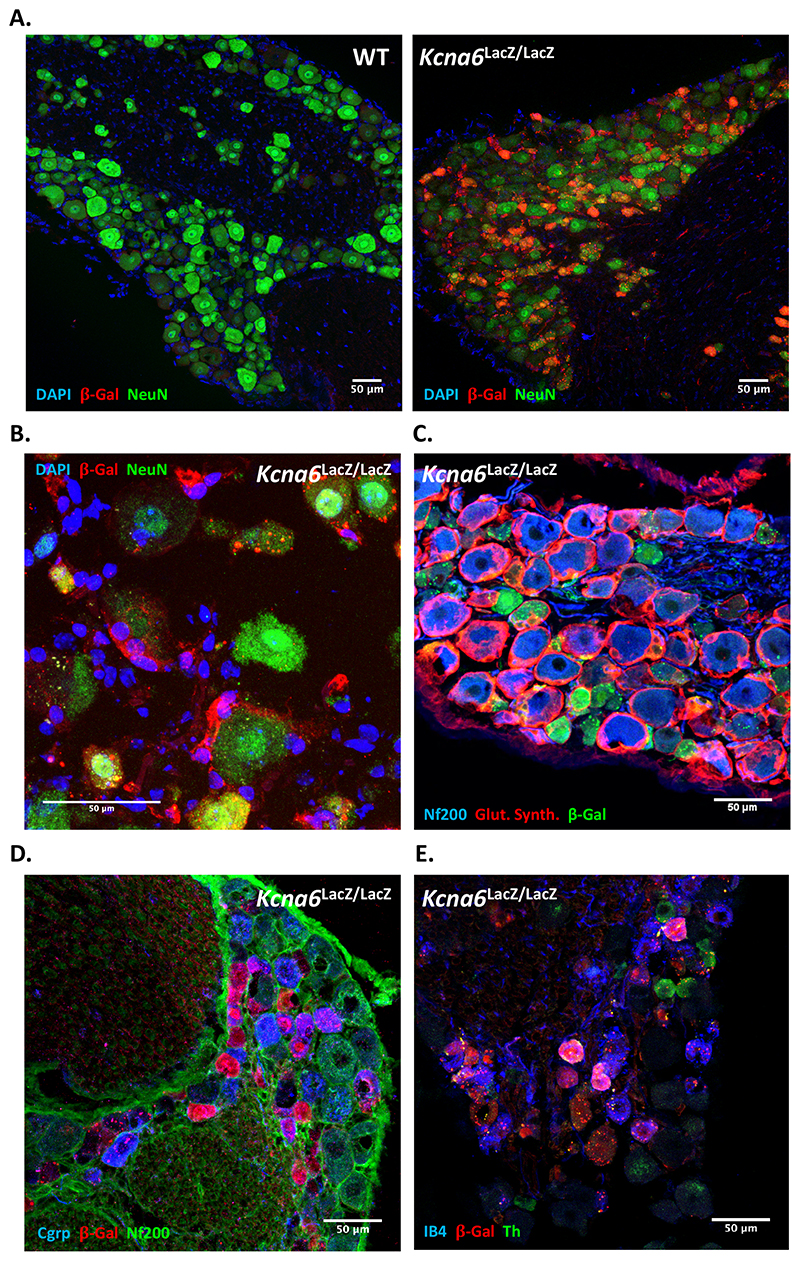
LacZ (β-galactosidase) expression in *Kcna6^lacZ/lacZ^* DRG **(A)** *Kcna6^lacZ^* carriers, but not wild-type littermates, express β-galactosidase under the Kcna6 promoter, detectable in DRG cells by immunostaining. **(B)** In addition to prominent β-galactosidase immunoreactivity in small neurons, staining revealed LacZ-expressing satellite glia ensheathing DRG neurons, confirmed with an antibody raised against satellite glial marker, glutamine synthetase **(C)**. **(D, E)** The Cgrp+ (± Nf200+), IB4+ and Th+ populations were all positive for β-galactosidase. Scale bars = 50μm

Using a chromogenic *in situ* hybridisation protocol (ACD Inc.) we visualised *Kcna6* expression in wild-type mouse L4 DRG neurons (Fig. 1A). Overall *Kcna6* expression was higher in smaller versus larger DRG neuronal populations. Across all cells examined from 4 animals, there is a significant negative correlation between mean *Kcna6* pixel intensity and cell area (Fig. 1B.i.), and across biological replicates small-diameter neurons had significantly greater expression than large-diameter neurons (Fig. 1B.ii.). We quantified *Kcna6* expression in 5 immunohistochemically defined subpopulations of sensory neurons – namely, myelinated neurons expressing neurofilament 200 (Nf200); peptidergic nociceptors expressing calcitonin gene-related peptide (Cgrp); C-low threshold mechanoreceptors expressing tyrosine hydroxylase (Th^+^ C-LTMRs); and non-peptidergic nociceptors binding to isolectin B4 (IB4). Overlap between the Nf200^+^ and Cgrp^+^ population demarcates the fifth subpopulation of myelinated peptidergic nociceptors. Signal from the *in situ* hybridisation was detected significantly above background fluorescence in nociceptive populations but not in the Th^+^ or Nf200^+^/Cgrp^-^ populations (Fig. 1C). The strongest expression of *Kcna6* was observed in myelinated peptidergic neurons (Nf200^+^/Cgrp^+^). We also noted *Kcna6* expression in satellite glial cells ensheathing DRG neurons, as identified by staining for glutamine synthetase (Fig. 1A, Fig. 2).

### Kcna6^lacZ^ knock-out mice exhibit altered nociceptive behaviour

**Figure 3:**
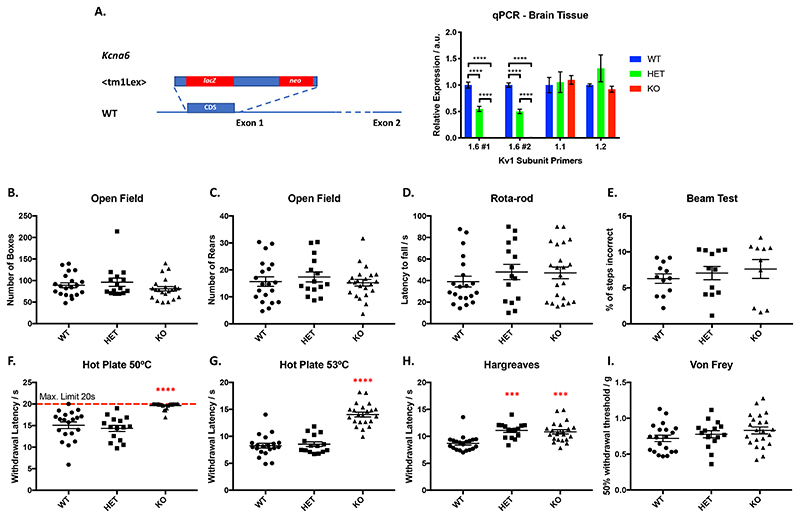
*Kcna6^lacZ^* mice are hyposensitive to noxious heat **(A)** The <tm1Lex> vector, containing a *lacZ* cassette, is introduced to the coding sequence (CDS) of *Kcna6* exon 1 by homologous recombination, resulting in *Kcna6* knock-out as confirmed by RT-qPCR (a.u. = arbitrary units relative to wild-type expression) on brain RNA extract using 2 sets of primers. Expression of two other Kv1 subunits (Kv1.1 and Kv1.2) is unaffected by *Kcna6* knock-out. n = 4 WT, 3 HET, 4 KO, **** = p<0.0001 (Ordinary One-Way ANOVA with Tukey’s post hoc multiple comparisons test). **(B-E)** *Kcna6^lacZ^* ^/+^ and ^*lacZ/lacZ*^ mice perform normally on open field and motor function tests. **(F-H)** *Kcna6^lacZ/lacZ^* mice are hyposensitive on 50°C and 53°C hot plate assays, while both heterozygous and homozygous mutants are hyposensitive on the Hargreaves radiant heat assay. **(I)** Neither groups differ from wild types in their response to mechanical stimulation by Von Frey hairs. Data points represent individual animal values, plotted with group mean ± SEM. n = 20 *Kcna6* ^+/+^ (11 male, 11 female), 15 *Kcna6^lacZ^* ^/+^ (13 male, 2 female), 22 *Kcna6^lacZ/lacZ^* (11 male, 11 female), apart from the beam test **(E)** n = 12 *Kcna6* ^+/+^ (6 male, 6 female), 12 *Kcna6^lacZ^* ^/+^ (10 male, 2 female), 10 *Kcna6^lacZ/lacZ^* (6 male, 4 female). *** = p<0.001, **** = p<0.0001 versus *Kcna6* ^+/+^ and *Kcna6^lacZ^* ^/+^ **(F,G)** or versus *Kcna6* ^+/+^ **(H)** (One-Way ANOVA with Tukey’s post hoc multiple comparisons test)

In order to investigate the contribution of Kv1.6 subunits to pain-like behaviour in mice, we procured a *Kcna6* knock-out mouse produced by homologous recombination with a <tm1Lex> cassette containing a *lacZ* reporter allele and neomycin phosphotransferase, henceforth termed *Kcna6^lacZ^* (MMRRC stock number: 011729-UCD). We confirmed expression of LacZ protein (β-galactosidase) by immunohistochemical detection in appropriate DRG populations and satellite glia (Fig. 2), and complete loss of *Kcna6* mRNA in brain tissue by quantitative reverse-transcriptase PCR (RT-qPCR) (Fig. 3A).

Behavioural phenotyping of this knock-out strain included an array of sensorimotor assays. Hetero- or homozygous *Kcna6^lacZ^* mice performed comparably to wild-type littermates on open field assays and motor co-ordination tasks (Fig. 3B-E). Homozygous *Kcna6^lacZ/lacZ^* knock-outs had a striking deficit in noxious thermal sensitivity on both hot plate (50°C and 53°C) and Hargreaves assays, whereas heterozygous littermates were hyposensitive only to stimulation via the Hargreaves test (Fig. 3F-H). Responsiveness to light punctate mechanical stimuli via Von Frey hair application was unaltered in either genotype compared to wild-type littermates (Fig. 3I).

### Kcna6^lacZ^ mice develop abnormal synapse-forming primary afferent terminals in the superficial dorsal horn

**Figure 4:**
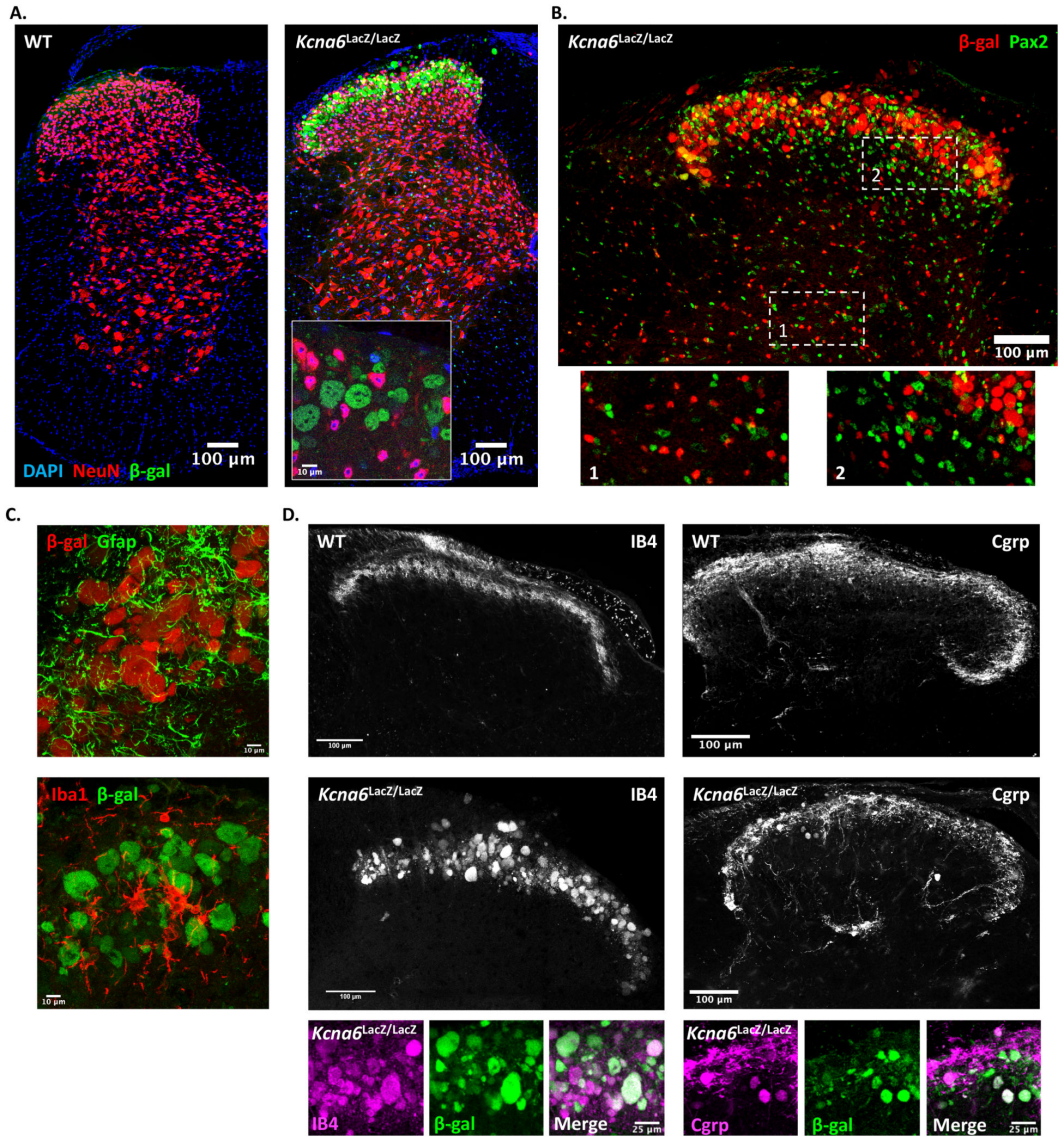
Abnormal anatomy in the *Kcna6^lacZ^* dorsal horn **(A)** LacZ (β-gal) staining in *Kcna6^lacZ^* homozygote spinal cord sections revealed striking abnormal, large profiles within superficial dorsal laminae. LacZ immunoreactivity also co-localised with smaller NeuN-negative, DAPI-positive profiles throughout grey and white matter. Inset: high power (63x objective) single z-slice image of abnormal swollen, vacuolated LacZ^+^ profiles in laminae I-II. Scale bar = 100μm (inset = 10μm). **(B)** Abnormal LacZ^+^ profiles do not co-localise with Pax2 (a marker of inhibitory interneurons), nor do smaller LacZ^+^ profiles in deeper grey and white matter. Scale bar = 100μm. **(C)** In superficial laminae, LacZ does not colocalise with markers of astrocytes (GFAP) or microglia (IBA1), but glial activation is often observed in close apposition to the abnormal LacZ^+^ profiles. Scale bar = 10μm. **(D)** Markers of non-peptidergic (IB4) and peptidergic (Cgrp) nociceptive populations known to terminate in superficial laminae show strong co-localisation with enlarged profiles in *Kcna6^lacZ^* homozygote spinal cord sections. Scale bars = 100μm (inset = 25μm).

**Figure 5:**
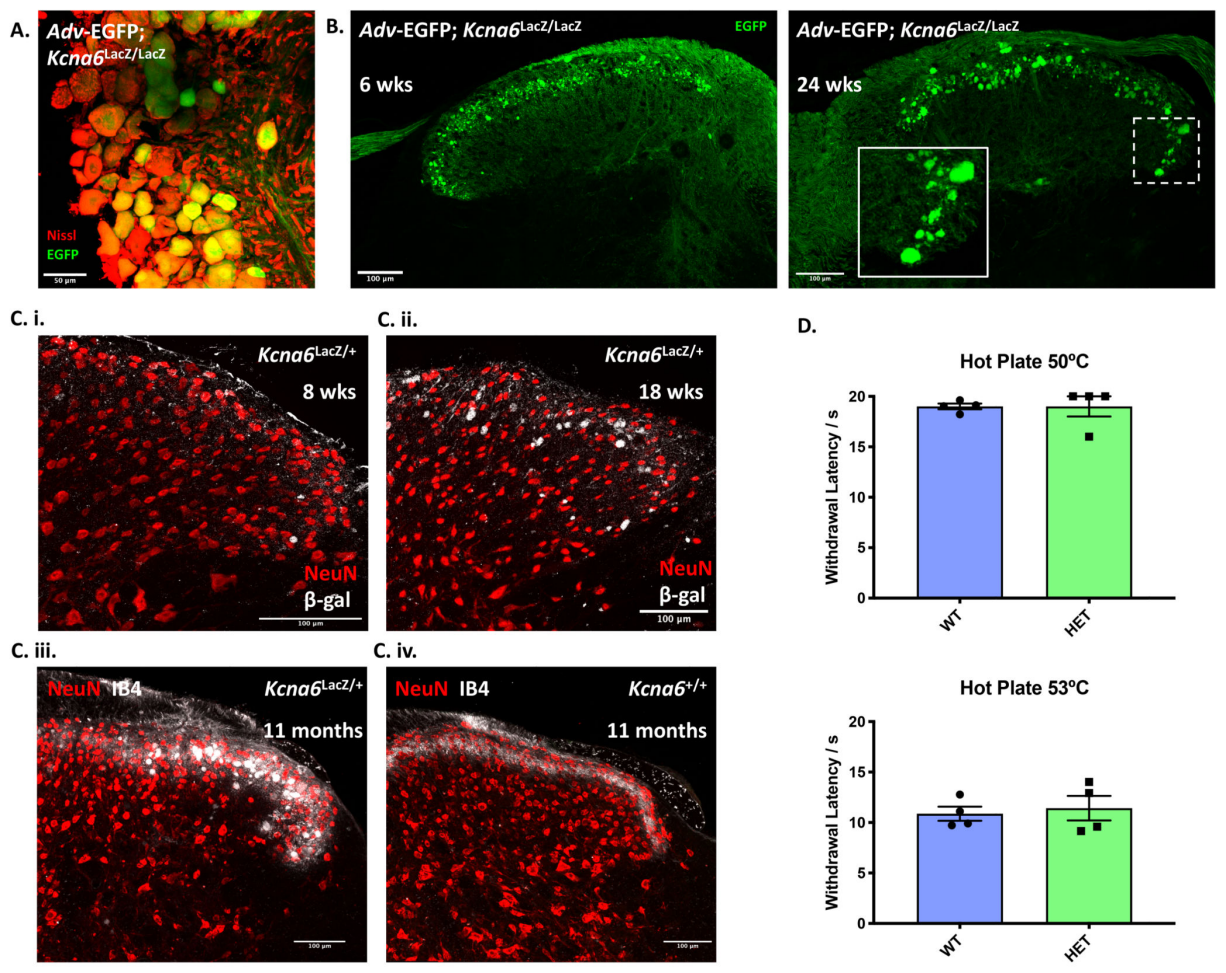
Degeneration of *Kcna6^lacZ^* primary afferents is progressive and gene dose-dependent **(A)** An *Advillin*-driven EGFP reporter mouse was crossed to the *Kcna6^lacZ/lacZ^* homozygotes to label DRG primary afferents marked by Nissl staining. Scale bar = 50μm **(B)** EGFP co-localized with the abnormal spinal profiles, confirming that they are primary afferent in origin. The pathology was present by 6 weeks postnatally, but also appeared to worsen with age. Scale bar = 100μm **(C)** *Kcna6^lacZ^* ^/+^ heterozygotes had a late-onset, slowly progressing pathology over a period of 11 months, evidenced by LacZ **(C.i-ii.)** or IB4 **(C.iii**.) staining, whereas wild-types were unaffected at 11 months **(C.iv.)**. Scale bar = 100μm **(D)** At 11 months, despite developing primary afferent terminal pathology, *Kcna6^lacZ^* ^/+^ heterozygote mice did not display any behavioural hyposensitivity to noxious heat compared to wild-type age-matched littermate controls. n = 4 *Kcna6* ^+/+^, 4 *Kcna6^lacZ/+^*(2 male, 2 female per genotype). p = 0.8451 (50°C) and 0.7273 (53°C) by unpaired, two-tailed t-test.

The most striking finding was a gross anatomical abnormality in the spinal dorsal horn of *Kcna6^lacZ^* mice. These abnormalities relate to the manipulation of the *Kcna6* locus, since wild-type littermates were anatomically normal and the abnormal structures in *Kcna6^lacZ/lacZ^* dorsal horn were intensely immunoreactive for the *lacZ* gene product β-galactosidase/LacZ (Fig. 4A-D). Specifically, we identified that central axonal projections of primary afferent neurons had swollen features in superficial as well as deep dorsal horn laminae. These abnormal swellings did not co-localise with neuronal (NeuN, Pax-2) or glial (Gfap – astrocytes, Iba1 – microglia) markers (Fig. 4A-C). There was, however, immunohistochemical co-localisation with IB4 and Cgrp (Fig. 4D), and interbreeding *Kcna6^lacZ/lacZ^* mice with *Advillin*-EGFP reporter mice (MMRRC stock number: 034769-UCD) demonstrated co-localisation of EGFP (Fig. 5A,B), confirming their primary afferent origin. The abnormal profiles were detected in both male and female *Kcna6^lacZ/lacZ^* mice. Some β-galactosidase staining distinct from the primary afferent terminals was detected throughout the grey and white matter, occasionally co-localising with Pax-2 (Fig. 4A,B). Since this LacZ reporter is driven by the endogenous *Kcna6* promoter, this likely reflects known low-level expression of *Kcna6* in some excitatory and inhibitory interneuron populations (Häring et al., 2018; Zeisel et al., 2018), or reported *Kcna6* expression in mouse and rat mature and progenitor oligodendrocytes (Attali et al., 1997; Chittajallu et al., 2002) and astrocytes (Smart et al., 1997; Zhu et al., 2014).

**Figure 6:**
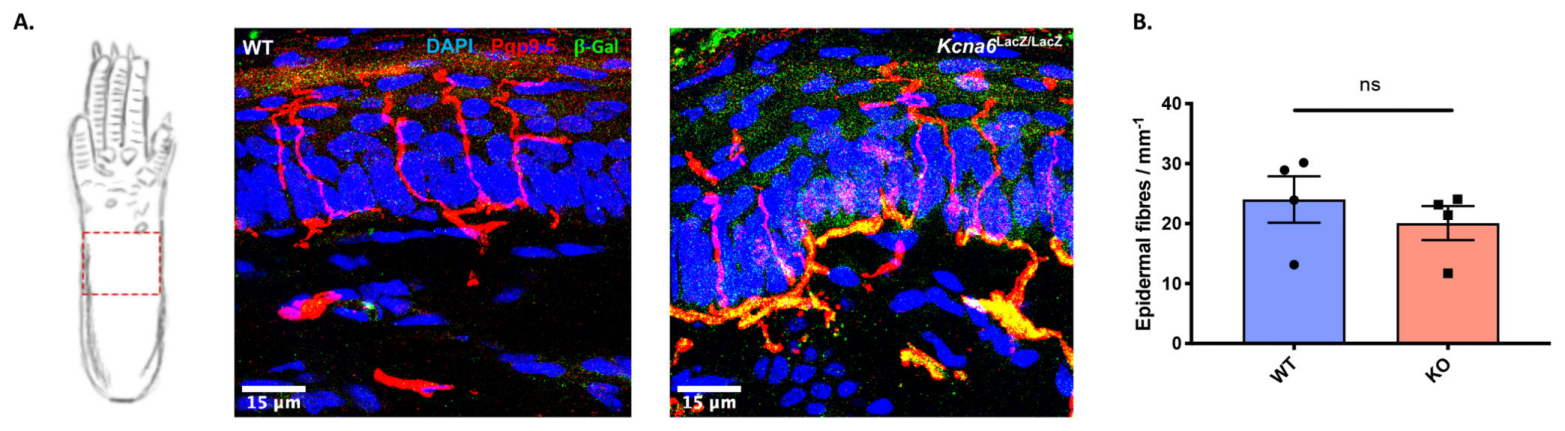
Intraepidermal nerve fibre morphology and density are unaltered in *Kcna6^lacZ/lacZ^* mice **(A)** Representative images of epidermal innervation from the indicated hind paw region of *Kcna6^lacZ/lacZ^* mice and wild type littermates. In knock-out mice, β-galactosidase immunoreactivity colocalises with the pan neuronal marker Pgp9.5 in the dermal nerve plexus. Scale bar = 15μm **(B)** Nerve fibre density in the epidermis did not differ significantly between knock-out and wild type mice. Data presented as mean ± SEM, plus mean values from individual animals. n = 4 *Kcna6* ^+/+^, 4 *Kcna6^lacZ/lacZ^* (2 male, 2 female per genotype). p = 0.4427 by unpaired, two-tailed t-test.

**Figure 7:**
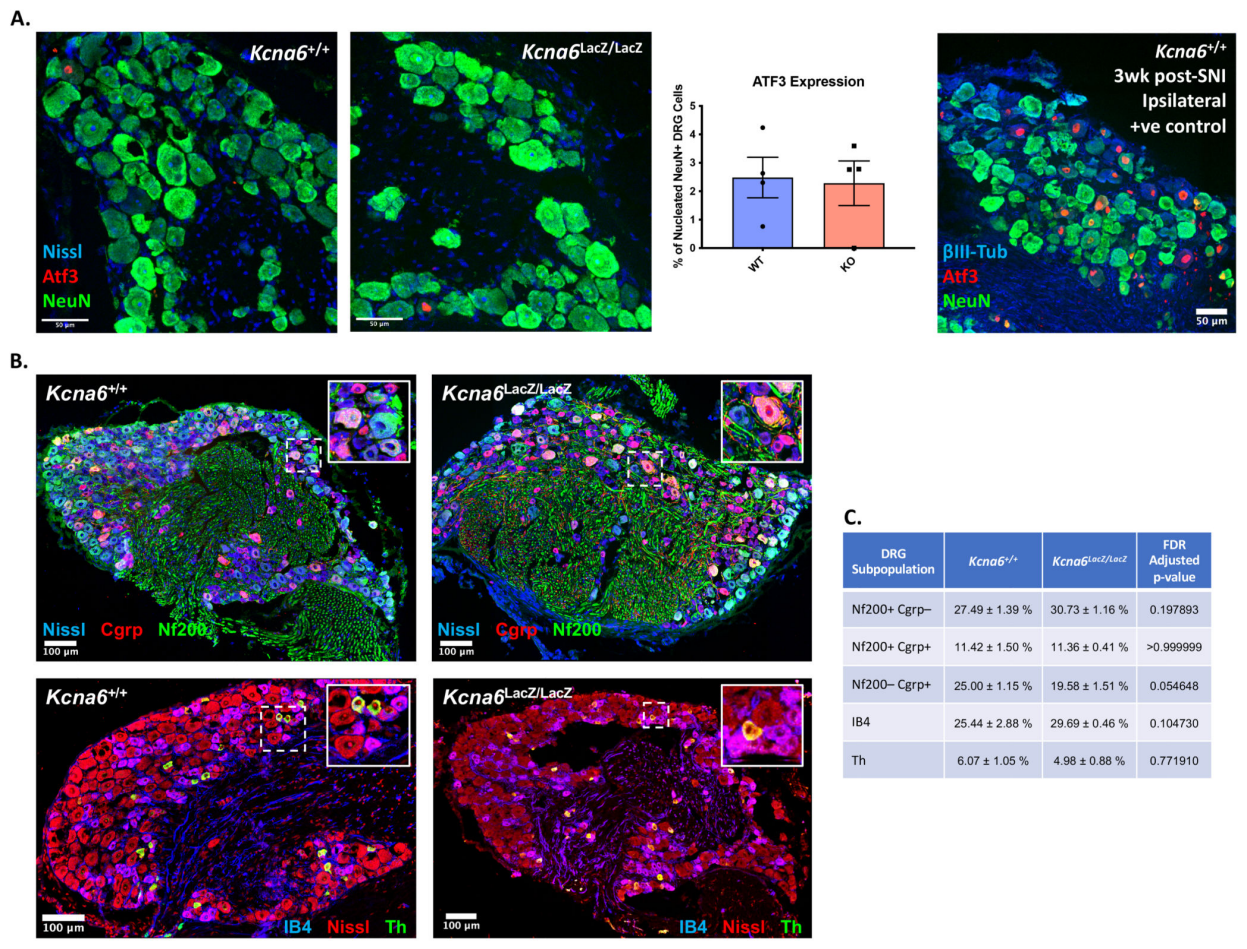
*Kcna6^lacZ/lacZ^* mice do not exhibit signs of cellular injury to or loss of DRG subpopulations **(A)** Despite pathology of primary afferent terminals, a lack of Atf3 upregulation suggests that injury-related gene-expression changes are not initiated in *Kcna6^lacZ/lacZ^* DRGs. A positive control image is provided from a wild type mouse that underwent Spared Nerve Injury. Scale bar = 50μm **(B)** Representative images of whole dorsal root ganglia sections *Kcna6^lacZ/lacZ^* mice and wild type littermates. Inset: magnification of dashed region of interest. Sections were stained for Nf200 and Cgrp, or IB4 and Th, with a fluorescent Nissl counterstain (Neurotrace). Scale bar = 100μm **(C)** Mean ± SEM percentages for each DRG subpopulation marker relative to Neurotrace (Nissl)-positive neurons. There is no significant difference between *Kcna6^lacZ/lacZ^* mice and wild-type littermates. Adjusted p-values comparing genotypes are indicated from multiple unpaired t-tests, performed with 5% False Discovery Rate using the Benjamini, Krieger and Yekutieli two-stage set-up method. n = 4 *Kcna6* ^+/+^, 4 *Kcna6^lacZ/lacZ^* (2 male, 2 female per genotype).

Six-week-old *Kcna6^lacZ/lacZ^; Advillin*-EGFP mice presented with clear swollen terminals, although the profiles were much reduced in size compared to at 24 weeks (Fig. 5B). Meanwhile, heterozygous *Kcna6^lacZ^* ^/+^ mice appeared to have a degree of protection against the development of this central terminal pathology but the phenotype progressed with increasing age (Fig. 5C.i-iii.). No evidence of degeneration was found in wild-type littermates at 11 months old (Fig. 5C.iv.). Compared to age-matched wild-types there appeared to be no accompanying thermal hyposensitivity in a small cohort of these aged *Kcna6^lacZ^* ^/+^ heterozygotes (Fig. 5D) even though these animals developed a structural abnormality – albeit to a lesser degree than the homozygotes. This pathology was only detected in the central terminals of *Kcna6^lacZ/lacZ^* primary afferents; morphology and counts of intra-epidermal nerve fibre density (IENFD) in the hind-paw skin were normal (Fig. 6). Pathology to sensory neuron terminals did not initiate upregulation of neural injury marker, Atf3 (Tsujino et al., 2000), in *Kcna6^lacZ/lacZ^* DRG neurons relative to wild-type littermates and proportion of Atf3^+^ DRG neurons in both genotypes was far lower than positive control DRG tissue sampled from wild-type mice after Spared Nerve Injury (Decosterd and Woolf, 2000) (Fig. 7A). There was also no obvious loss of neurons in any DRG subpopulation (Fig. 7B).

**Figure 8:**
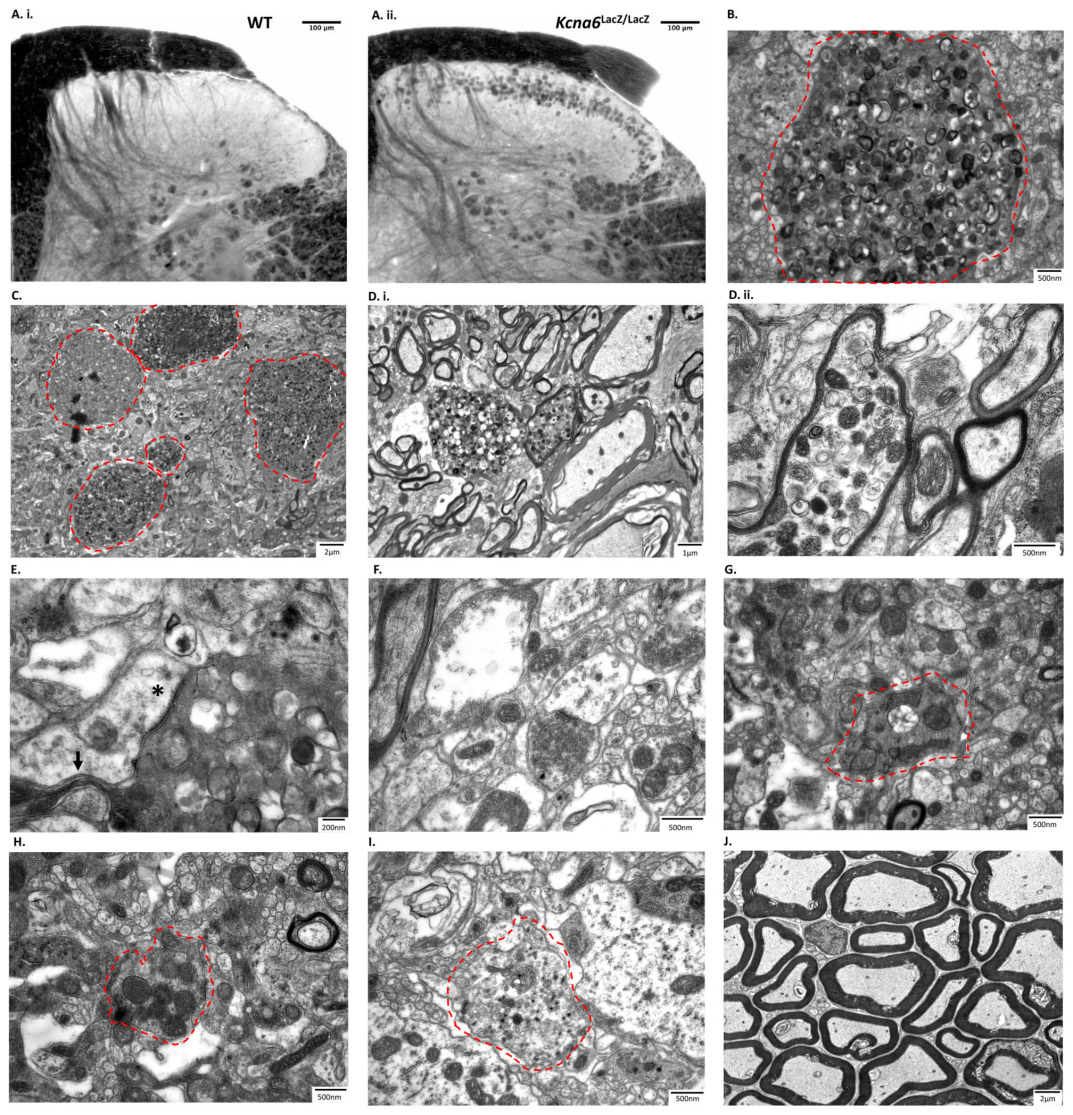
Ultrastructural analysis of degenerative *Kcna6^lacZ^* primary afferents **(A)** 60μm thick osmicated sections showing normal anatomy in wild-type tissue (A. i.), and lipid-rich profiles in the superficial *Kcna6^lacZ/lacZ^* dorsal horn (A. ii.). **(B, C)** Swollen terminals (red dashed areas) vary in size and electron density and contain many small vesicular components. **(D)** Some of the abnormal vesicle-containing profiles originate from myelinated afferents in Lissauer’s tract. **(E)** Numerous profiles were observed adjacent to postsynaptic dendrites (asterisk) and sprouting intervaricose axons (arrow) but lacked presynaptic neurotransmitter vesicles. **(F)** Some normal synapses were observed. **(G)** Presumed type I glomerular central boutons (C_1_) originating from IB4^+^ neurons appeared abnormal, as did **(H)** presumed type II glomerular central boutons (C_2_) and **(I)** peptidergic boutons containing dense-cored vesicles. Red dashed areas indicate the abnormal afferent terminals. **(J)** Axons in the dorsal root were normal.

Transmission electron microscopy (TEM) was used to analyse these dorsal horn structures at nanoscale. The abnormal axon terminals identified previously were stained intensely by osmium when observed using light microscopy in *Kcna6^lacZ/lacZ^* tissue, suggesting the presence of a high concentration of lipids (Fig. 8A.i-ii.). TEM revealed densely packed, lipid-rich vesicular bodies contained within membrane-delimited profiles (Fig. 8B). Both the abnormal profiles and the vesicles within were highly variable in size and electron density (Fig. 8C). Some profiles were ensheathed by myelin, within or in close proximity to Lissauer’s tract (Fig. 8D.i-ii.). Others made clear synaptic contacts with adjacent dendrites containing post synaptic densities, and often appeared to send out multiple cytoplasmic projections from their swollen region – tentatively designated as inter-varicose axons (Fig. 8E).

While some normal presynaptic boutons (of unknown origin) could be found in the superficial laminae (Fig. 8F), there was a notable reduction of normal type I (C_1_) and type II (C_2_) glomerular central boutons, originating from IB4^+^ neurons and Aδ- or C-LTMRs, respectively (West et al., 2015; Gutierrez-Mecinas et al., 2016; Larsson and Broman, 2019). The few examples of central glomerular boutons appeared highly irregular in form and content (Fig. 8G-I). Type I central boutons (C_1_) are described as having “a dark small central terminal of indented contour with closely packed spherical vesicles of variable diameter and few mitochondria” (Ribeiro‐Da‐Silva and Coimbra, 1982), but the example in (Fig. 8G) lacks vesicles and is very abnormal. Some type II central boutons (C_2_) and peptidergic axonal boutons (containing dense-cored vesicles) were observed, although similarly these did not look normal and potentially included vesicular pathology (Fig. 8H-I). The dorsal root was unaffected (Fig. 8J), so the accumulation of membranous, vesicular bodies appeared to occur once afferents had penetrated the cord.

### Dorsal horn wide dynamic range neurons from Kcna6^lacZ^ mice have reduced electrophysiological responses to peripheral stimuli

We reasoned that the abnormality in primary afferent terminals was highly likely to underlie behavioural deficits in *Kcna6^lacZ/lacZ^* mice via impaired neurotransmission from first- to second-order afferent neurons. We therefore undertook electrophysiological analysis of the response of deep dorsal horn wide dynamic range neurons (WDRs) to hind paw stimulation.

**Figure 9:**
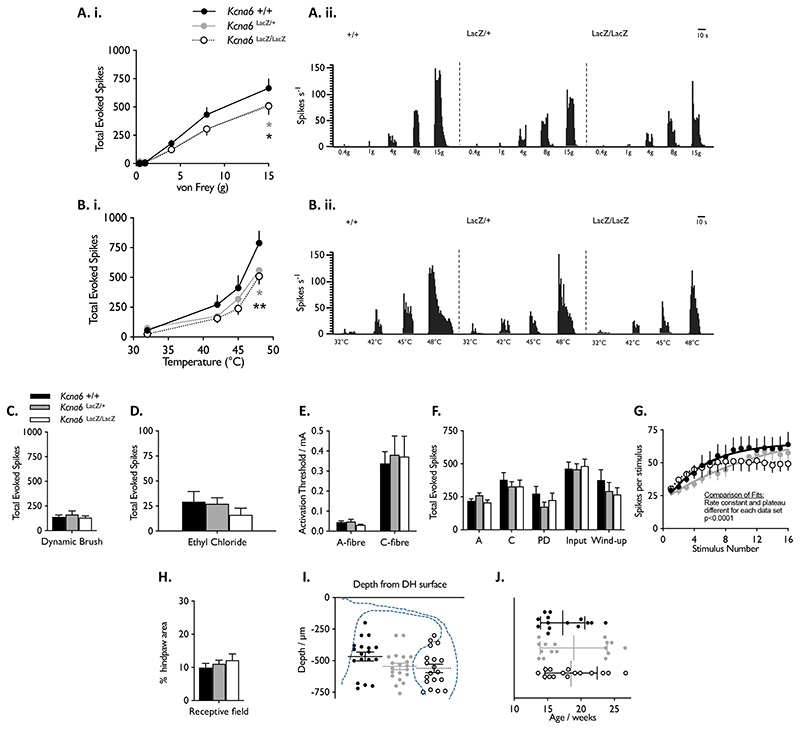
Electrophysiological characterisation of *Kcna6^lacZ^* WDR neurons **(A.i., B.i.)** Graded firing responses in wide dynamic range (WDR) neurons, evoked by mechanical (Von Frey) or hot water stimuli of increasing intensity applied to the hind paw. **(A.ii. B.ii)** Representative traces from single neurons. */** = p<0.05/0.01 vs WT (Two-Way RM ANOVA with Dunnett’s post hoc comparison test.) **(C, D)** Responses evoked by dynamic application of a painter’s brush, or droplets of evaporative cooling agents. * = p<0.05 (Ordinary One-Way ANOVA, Tukey’s post hoc test) **(E)** A- and C-fibre activation thresholds as determined by WDR response latency to electrical stimulation of the hind paw receptive field. **(F)** Total spikes attributed to A- or C-fibres, or post-discharge (PD) as determined by WDR response latency, and attributed to input vs. wind-up (see methods). **(G)** Firing responses to a train of 16 identical electrical stimuli at a frequency 0.5Hz. **(H-J)** Characterisation of receptive field size **(H)**, and WDR depth from the dorsal spinal cord surface **(I)**, and a graphic depicting the age of mice from which WDRs were sampled. Group data presented as mean ± SEM in all panels except **(J)** which is mean ± SD. **(A-D, H, I)** n = 19 neurons from 14 *Kcna6* ^+/+^ (7 male, 7 female), 21 neurons from 16 *Kcna6^lacZ^* ^/+^ (10 male, 6 female), 19 neurons from 15 *Kcna6^lacZ/lacZ^* (6 male, 9 female) mice. **(E-G)** n = 15 of these neurons per genotype had electrical properties characterised.

Wild-type, *Kcna6^lacZ^* ^/+^ and *Kcna6^lacZ/lacZ^* WDR firing rates were characterised after ‘natural’ stimulation by calibrated Von Frey filaments, graded temperatures of water, brush, and evaporative cooling. In all genotypes, the graded intensity of mechanical and thermal stimuli was coded in the WDR firing rate, as is expected of these neurons (Fig. 9A,B). However, at the noxious end of the mechanical and thermal repertoire there were significant deficits in spike generation in both *Kcna6^lacZ^* hetero- and homozygote mice following 15g Von Frey or 48°C heat stimulation (Fig. 9A,B). Responses to non-noxious mechanical brush stimulation did not differ between genotypes (Fig. 9C). Evaporative cooling with ethyl chloride did not elicit significantly different responses across genotypes (Fig. 9D).

Electrical activation thresholds for A- and C-fibre-evoked spikes were unchanged in hetero- or homozygous *Kcna6^lacZ^* animals (Fig. 9E) versus wild-types, nor were there statistical differences in the total evoked spikes by either fibre class after a train of 16 electrical stimuli to the receptive field on the paw (Fig. 9F). The number of spikes generated after the C-fibre latency window (termed ‘post-discharge (PD)’) was also not affected (Fig. 9F). While the total number of spikes attributed to ‘wind-up’ of WDRs (that is, the progressive increase in responsivity with each stimulus) was not different between genotypes (Fig. 9F), there were some considerable differences in the kinetics of wind-up between wild-types and *Kcna6^lacZ^* mutants. Specifically, WDR responses in wild-types followed a typical hyperbolic pattern, gradually reaching a maximal discharge rate towards the end of the 16-stimulus paradigm (Fig. 9G). Knock-outs had a similar initial rate of wind-up but reached a lower plateau, while wind-up in heterozygotes formed a much more linear relationship with increasing stimuli. Epidermal receptive fields providing input to WDRs were similar in size across all genotypes, as was the range of sampled neuron depths and the age of mice at the time of experiment (Fig. 9H-J).

### LacZ-negative Kcna6^-/-^ mice have milder behavioural phenotypes with no pathology in presynaptic primary afferent terminals

**Figure 10:**
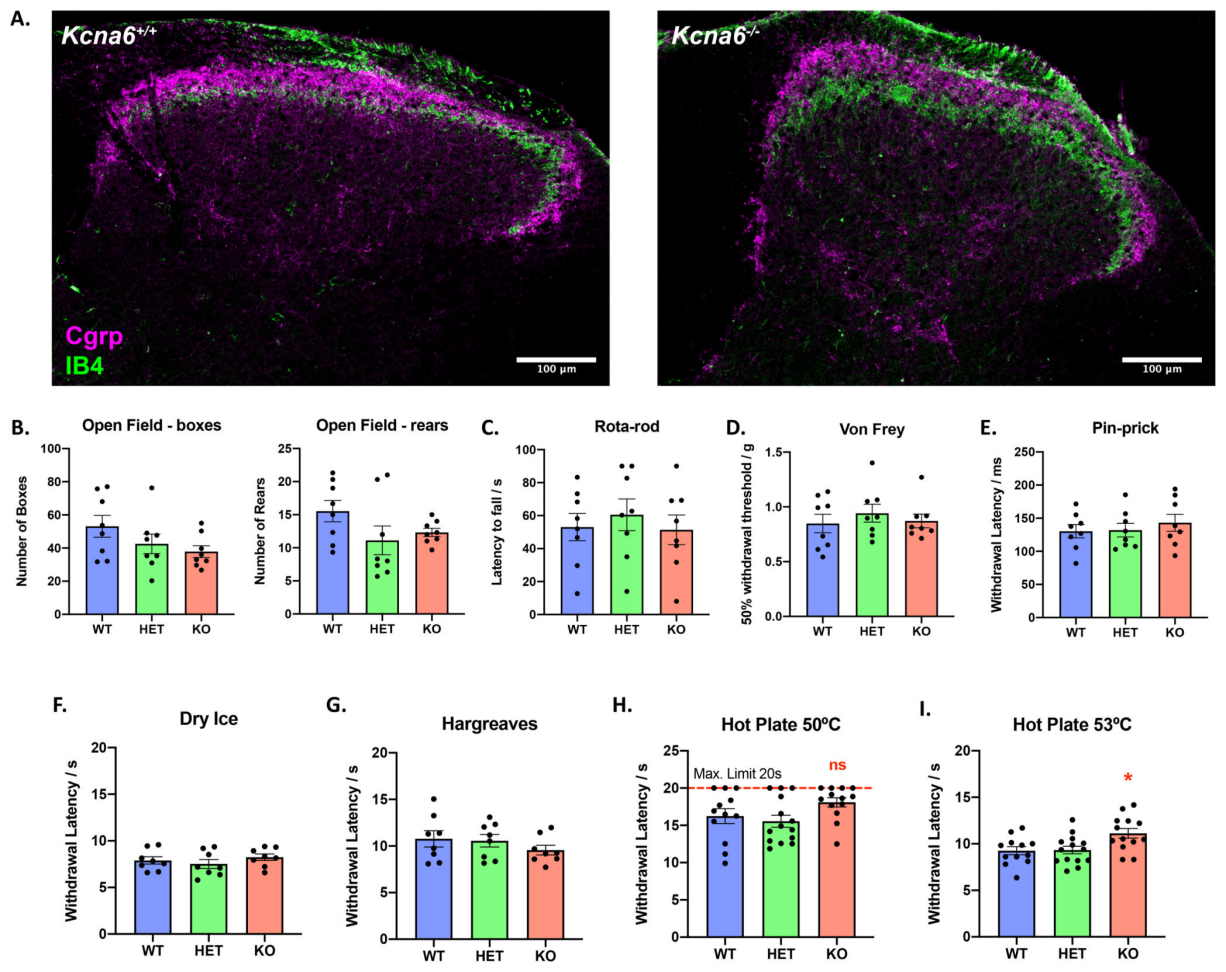
Anatomical and behavioural consequences of CRISPR-mediated *Kcna6* deletion independent of *lacZ* expression **(A)** Comparison of IB4^+^ and Cgrp^+^ central terminal staining in CRISPR-mediated *Kcna6* ^-/-^ homozygote knock-outs and wild-type littermates >15 weeks old reveals normal morphology. Scale bar = 100μm. **(B)** Measurement of exploratory behaviour (gridline crosses and rearing episodes) averaged across 3 baselines. **(C)** Motor performance on Rota-rod was unaffected in *Kcna6* ^+/-^ and *Kcna6* ^-/-^ mice. **(D-I)** Withdrawal threshold or latency to plantar hind-paw stimulation by Von Frey **(D)**, pin-prick **(E)**, dry ice **(F)**, Hargreaves **(G)** and hot plate **(H, I)**. *=p<0.05 vs. *Kcna6* ^+/+^ by One-Way ANOVA **(I)** with Dunnett’s multiple comparisons test. n = 8 *Kcna6* ^+/+^ (4 male, 4 female), 8 *Kcna6* ^+/-^ (4 male, 4 female), 8 *Kcna6* ^-/-^ (5 male, 3 female) **(B-G)**; n = 12 *Kcna6* ^+/+^ (4 male, 8 female), 14 *Kcna6* ^+/-^ (6 male, 8 female), 13 *Kcna6* ^-/-^ (6 male, 7 female) **(H, I)**; mean ± SEM

The degenerative phenotype affecting the central terminals of primary afferents in *Kcna6^lacZ/lacZ^* mice was unexpected and had not previously been reported in other *Shaker*-like potassium channel mutants. For comparison to the very unusual phenotype of the *Kcna6^lacZ^* strain, we characterised a different commercially available CRISPR-mediated constitutive *Kcna6* knock-out mouse (MMRRC stock number: 042391-JAX), containing no exogenous *lacZ* cassette. Spinal cord tissue was collected from these homozygous *Kcna6* ^-/-^ mice aged >15 weeks, by which time point dorsal horn pathology had fully developed in *Kcna6^lacZ/lacZ^* mice. However, there was no sign of a similar effect on central primary afferent terminals; the superficial dorsal horn neuropil appeared normal, with dense peptidergic and non-peptidergic fibre reactivity in lamina II (Fig. 10A).

Responses to a wide range of sensory (noxious and non-noxious) stimuli across multiple modalities were assessed, and mice were also challenged with multiple motor tasks. Performance across tasks such as the open field and rota-rod challenges was comparable between *Kcna6* ^-/-^, *Kcna6* ^+/-^, and *Kcna6* ^+/+^ littermate mice (Fig. 10B,C). Responses to punctate mechanical stimulation by Von Frey hairs and more noxious pin-prick were also similar (Fig. 10D,E), as well as responses to noxious cooling of the hind-paw with the dry ice test (Fig. 10F) (Brenner et al., 2012). Compared to the phenotype of *Kcna6^lacZ^* knock-outs, the thermal hyposensitivity of this *Kcna6* ^-/-^ cohort was much milder, with no discernible difference in withdrawal latency to the Hargreaves test or a 50°C hot plate (Fig. 10G,H). However, with more intense noxious hot plate stimulation at 53°C, the *Kcna6* ^-/-^ cohort had a mildly but significantly delayed response time (Fig. 10I).

### Nav1.8^Cre/+^-dependent expression of LacZ is sufficient to induce presynaptic pathology in primary afferent terminals

The absence of primary afferent degeneration in *Kcna6* ^-/-^ mice suggested that the expression of exogenous LacZ was responsible for the pathology identified in the dorsal horn of *Kcna6^lacZ^* mice. However, it remained possible that this phenomenon arose due to: a) the neomycin resistance cassette also present in the <tm1Lex> alleles of *Kcna6^lacZ^* mice (Fig. 3A); or b) an unanticipated interaction between the loss of Kv1.6 subunits and expression of LacZ. To seek definitive evidence that LacZ expression alone is intrinsically harmful to the central terminals of nociceptive primary afferent neurons, we used a Nav1.8 promoter-driven Cre recombinase (Nassar et al., 2004; Stirling et al., 2005) to induce expression of exogenous LacZ (Soriano, 1999) on a *Kcna6* ^+/+^ background in primary afferent nociceptors. The breeding of Nav1.8^Cre/+^ and ROSA26^*lacZ/lacZ*^ produced Nav1.8^Cre/+^;ROSA26^*lacZ*/+^ compound heterozygotes expressing LacZ protein in Nav1.8^+^ neurons, which overlap considerably with *Kcna6* ^+^ neurons in the DRG (Usoskin et al., 2015; Zeisel et al., 2018; Zheng et al., 2019).

**Figure 11:**
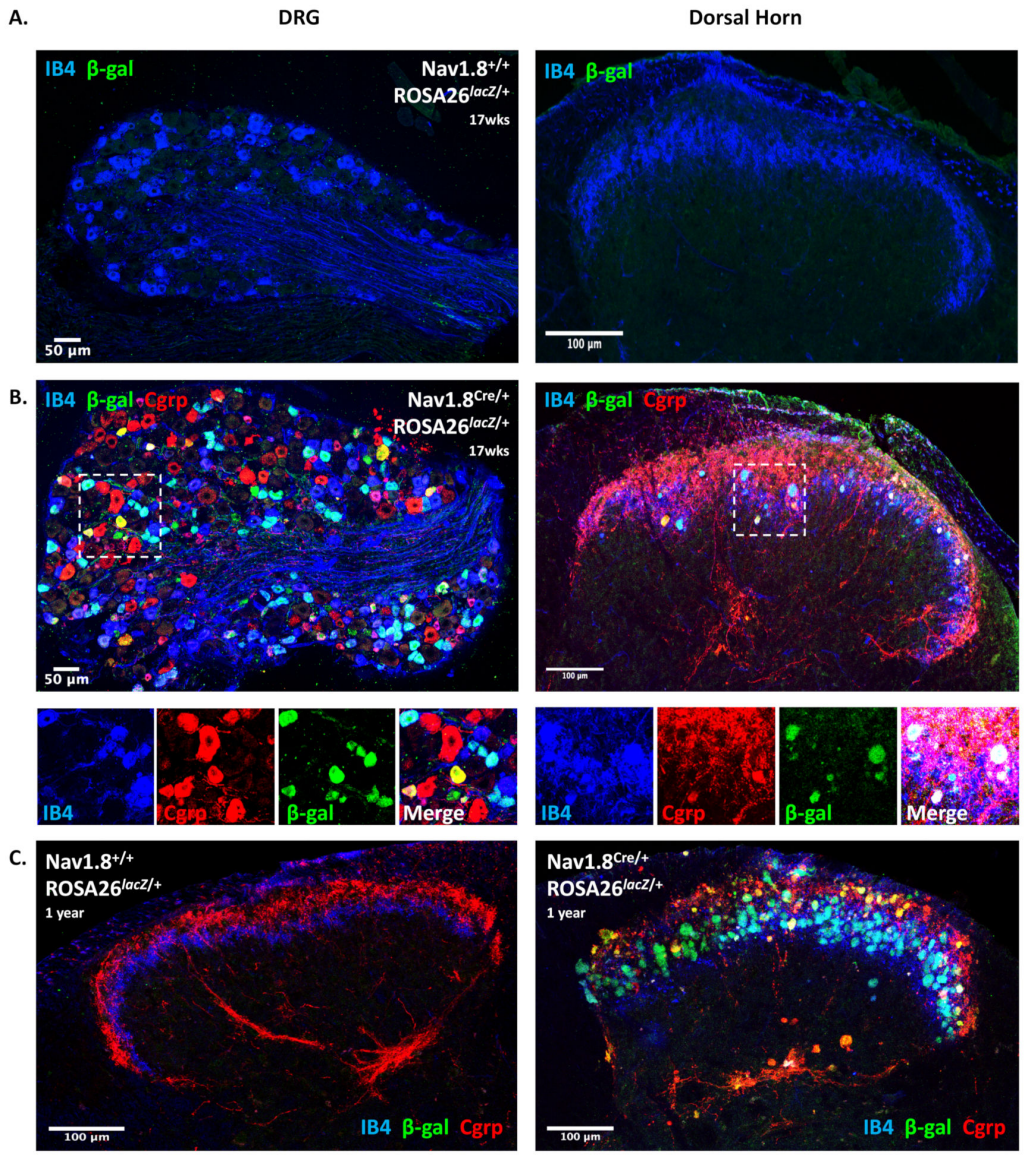
Anatomical consequences of nociceptor-specific LacZ expression independent of *Kcna6* deletion **(A)** No Cre-mediated LacZ expression in Nav1.8 Cre-negative mice >17 weeks old expressing the ROSA26^*lacZ*^ reporter allele. IB4^+^ terminals in the dorsal horn appear normal in these mice despite expressing *neo* globally. **(B)** In >17 weeks old mice carrying one ROSA26^*lacZ*^ allele and one Nav1.8 Cre allele, *lacZ* is expressed in small diameter, IB4^+^ and CGRP^+^ DRG neurons. Swollen nociceptor terminals containing β-galactosidase are clearly visible in the superficial laminae. Images in **(A)** and **(B)** are representative paired DRG and dorsal horn sections from the same animals. **(C)** One-year-old Cre negative mice remain phenotypically normal. In 1-year-old Nav1.8^Cre/+^;ROSA26^*lacZ*/+^ mice, dorsal horn pathology was much more extensive, including in Cgrp^+^ afferents projecting to deeper laminae. Scale bars = 50μm (DRG), 100μm (Dorsal Horn).

Neither LacZ protein, nor dorsal horn abnormalities, were detected by immunohistochemistry in 17-week old Cre-negative Nav1.8^+/+^;ROSA26^*lacZ*/+^ mice which, without the presence of Cre recombinase, express a neomycin resistance cassette globally (Fig. 11A). This suggests that exogenous neomycin phosphotransferase was not responsible for primary afferent terminal degeneration in *Kcna6^lacZ^* mice. In Nav1.8 Cre-positive mice also carrying the ROSA26^*lacZ*^ allele, exogenous LacZ expression was detected and abnormal LacZ^+^ presynaptic terminals were once again visible within the dorsal horn, co-localising with IB4 and Cgrp staining (Fig. 11*B*). These were however less numerous than in the *Kcna6^lacZ^* animals at the equivalent age. Nav1.8^Cre/+^;ROSA26^*lacZ*/+^ mice had far more extensive evidence of primary afferent degeneration in the dorsal horn at approximately 1 year of age, including lesions of axons within the attached dorsal root and those projecting into deeper laminae (Fig. 11C). Nav1.8 Cre-negative littermates demonstrated normal dorsal horn morphology at this time point.

**Figure 12:**
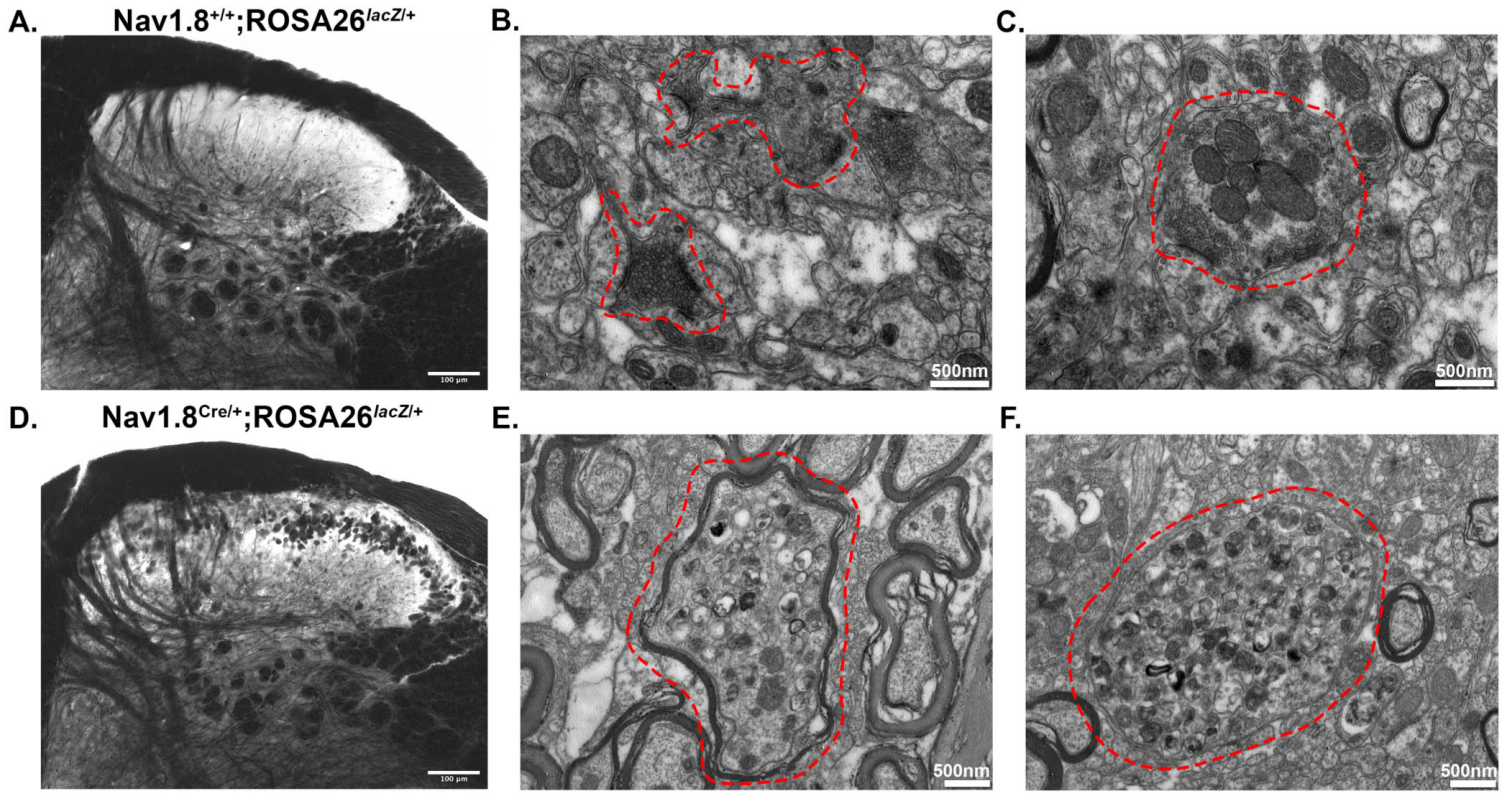
LacZ causes similar ultrastructural lipid abnormalities to nociceptor terminals when expressed in Nav1.8-Cre neurons **(A, D)** Light micrographs of osmicated dorsal horn sections from Nav1.8^+/+^;ROSA26^*lacZ*/+^ and Nav1.8^Cre/+^;ROSA26^*lacZ*/+^ reveals that degenerative primary afferent terminals in Nav1.8-Cre positive mice are rich in lipid species, similarly to *Kcna6^lacZ/lacZ^* mice. Scale = 100μm. **(B, C)** TEM imaging of Nav1.8^+/+^;ROSA26^*lacZ*/+^ dorsal horn showing normal appearance of type I **(B)** and type II **(C)** glomerular central boutons (red dashed areas) forming multiple synaptic contacts in lamina II. Scale bar = 500nm. **(E, F)** TEM imaging of Nav1.8^Cre/+^;ROSA26^*lacZ*/+^ dorsal horn sections reveals axon terminals with similar ultrastructural morphological features to *Kcna6^lacZ/lacZ^* mice (red dashed areas), including numerous and varied endo/lysosomal-like structures within. Scale bar = 500nm.

**Figure 13:**
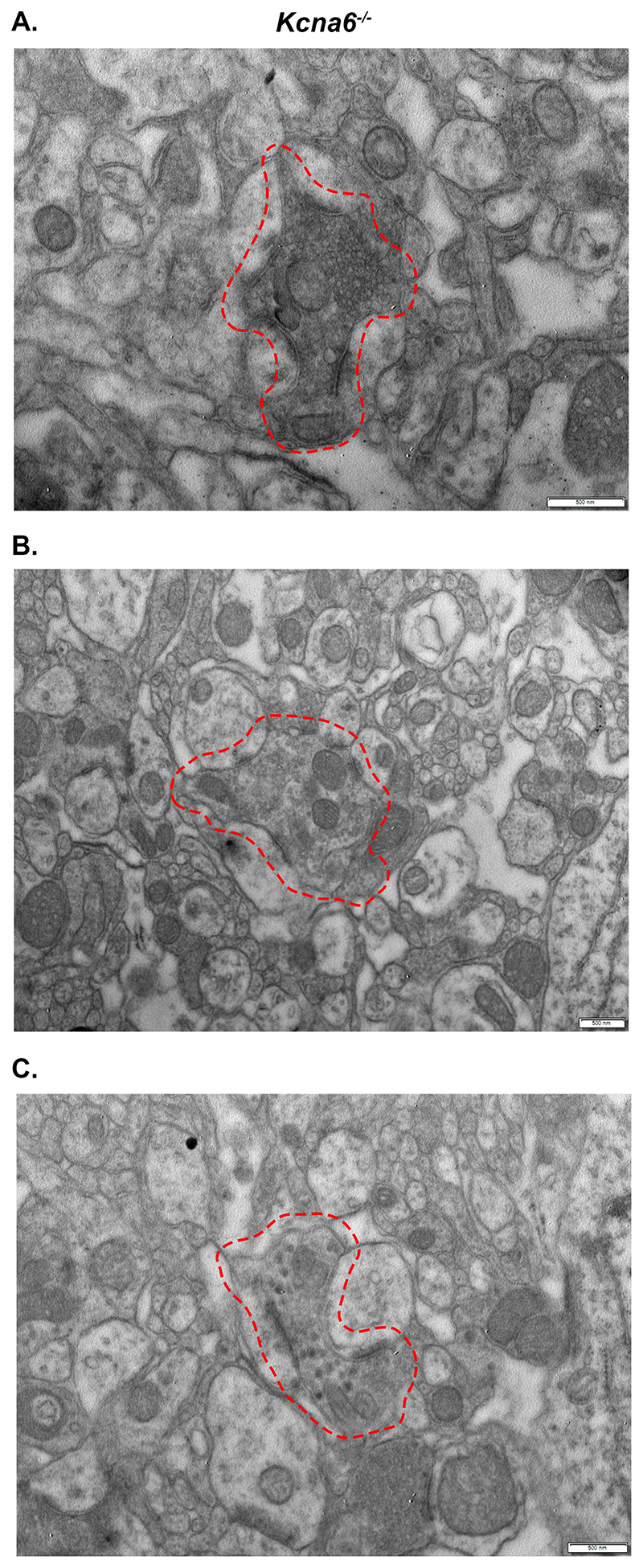
*Kcna6* ^-/-^ knock-out mice not expressing *lacZ* have normal morphological dorsal horn features **(A)** Normal appearance of a type I glomerular central bouton (red dashed area) in a *Kcna6* ^-/-^ mouse. Scale = 500nm. **(B)** Normal appearance of a type II glomerular central bouton (red dashed area) in a *Kcna6* ^-/-^ mouse. Scale = 500nm. **(C)** Normal appearance of a peptidergic bouton (red dashed area) containing dense-core vesicles that forms two asymmetrical synapses, in a *Kcna6* ^-/-^ mouse. Scale = 500nm.

Viewed with TEM, the ultrastructure of pathological Nav1.8^Cre/+^;ROSA26^*lacZ*/+^ afferents (Fig. 12) was similar to those of the *Kcna6^lacZ/lacZ^* mice (Fig. 8), while normal morphology of primary afferent terminals was observed in Nav1.8-Cre negative littermate controls (Fig. 12) and in *Kcna6* ^-/-^ knock-out mice not expressing *lacZ* (Fig. 13). Clearly, the presence of exogenous LacZ protein in DRG neurons is sufficient to cause age-dependent deterioration of primary afferent presynaptic boutons.

### Ganglioside metabolism is implicated in LacZ-mediated pathophysiology

**Figure 14:**
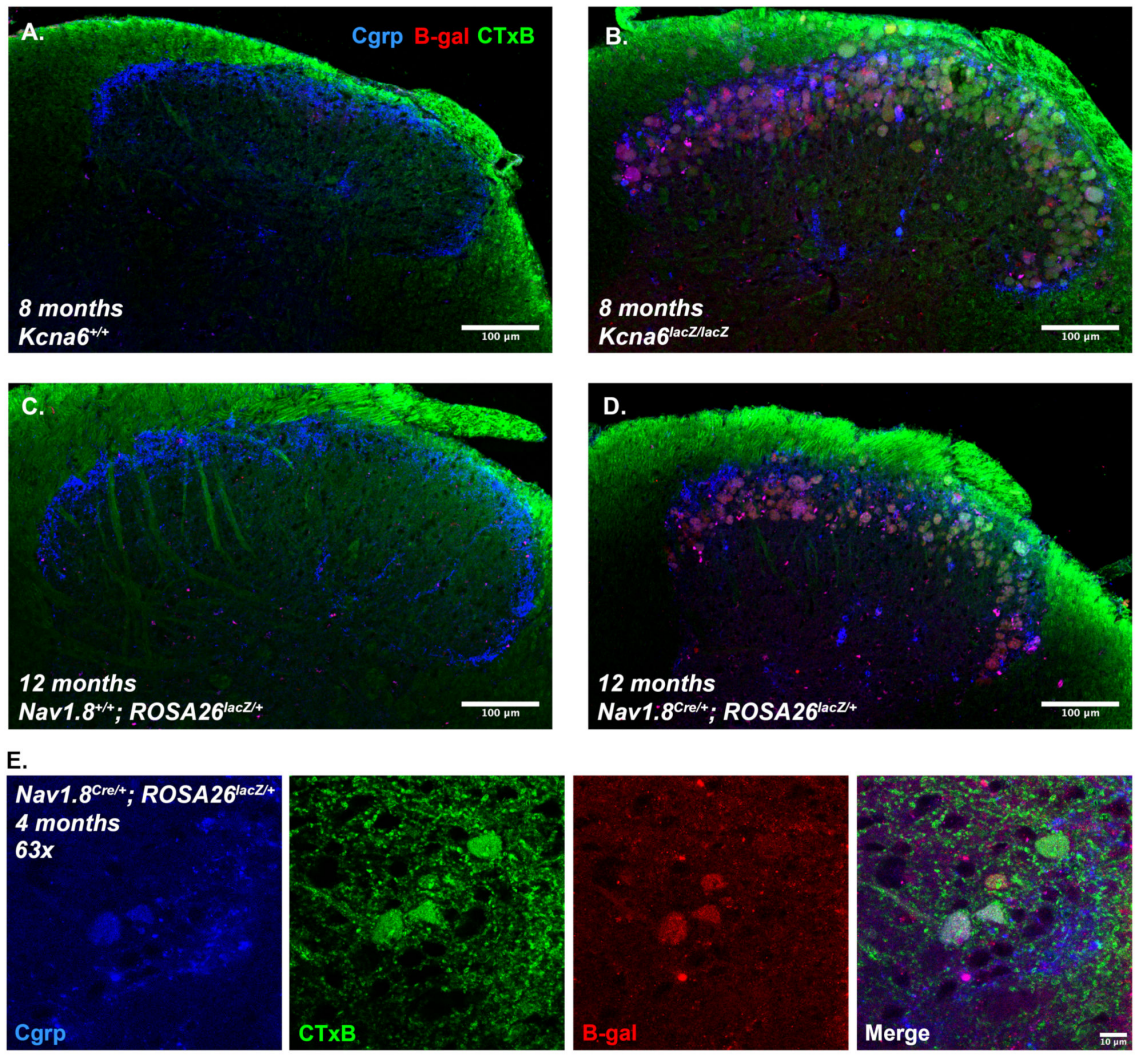
Accumulation of GM1 gangliosides in LacZ-affected primary afferent terminals **(A-D)** Staining for GM1 ganglioside with Alexa488-conjugated cholera toxin B (CTxB) reveals GM1 in primary afferent fibres in the mouse dorsal root, dorsal column white matter tracts, and dorsal horn grey matter. Additionally, in 8-month-old *Kcna6^lacZ/lacZ^* mice **(B)** or 12-month-old Nav1.8^Cre/+^;ROSA26^*lacZ*/+^ mice **(D)**, GM1 co-localised with aberrant afferent terminals marked by β-galactosidase and Cgrp immunostaining. Scale bars = 100μm. **(E)** High-power images of Nav1.8^Cre/+^;ROSA26^*lacZ*/+^ afferent terminals depict punctate CTxB staining in abnormal afferent terminals, co-localised with β-galactosidase and/or Cgrp.

**Figure 15:**
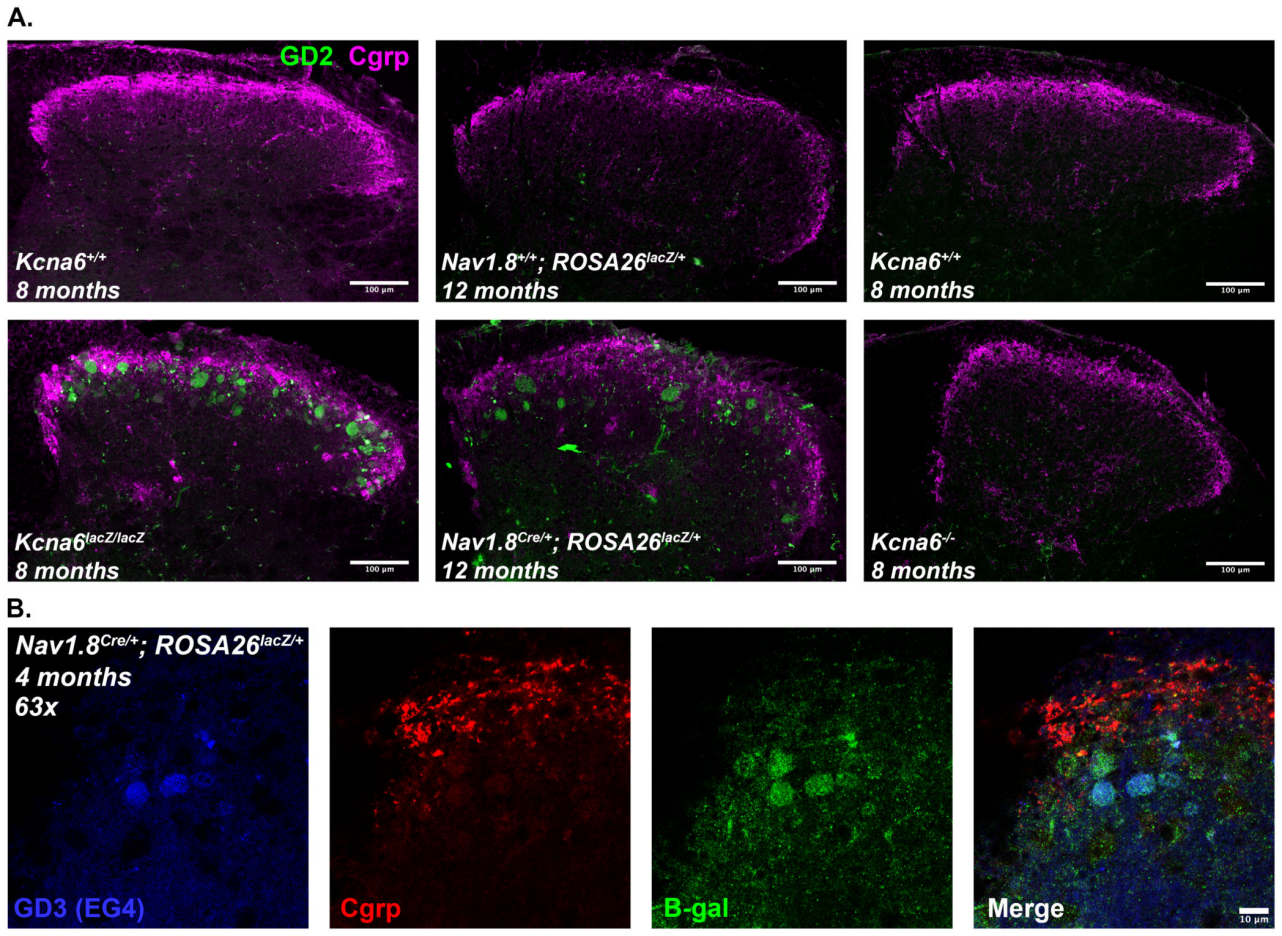
Multiple ganglioside species accumulate in LacZ-affected primary afferent terminals **(A)** Mouse monoclonal antibodies raised against ganglioside GD2 label degenerating nociceptor terminals in *Kcna6^lacZ/lacZ^* and Nav1.8^Cre/+^;ROSA26^*lacZ*/+^ mice, but not (non-LacZ) *Kcna6* ^-/-^ mice or littermate controls of any strain. Scale bars = 100μm. **(B)** Mouse anti-GD3 (EG4) antibodies also label GT1b/GQ1b, co-localising with some β-galactosidase positive terminals in lamina II of the superficial dorsal horn. Scale bar = 10μm.

LacZ is a bacterial enzyme with β-galactosidase activity (Juers et al., 2012), and is therefore well-placed to interfere with endo-lysosomal metabolism of complex gangliosides (sialic acid-containing glycosphingolipids), the major constituent of mammalian cell membranes. Neurons chiefly synthesise a- and b-series gangliosides including GM1, cleavage of which to GM2 is achieved throughout the endo-lysosomal pathway by GM1-β-galactosidase removing a terminal β-galactose residue. We hypothesised that overexpression of exogenous LacZ may result in accumulation of GM1, GM2, or other neuronal ganglioside species, and tested this using immunohistochemical techniques. GM1 is bound by cholera toxin-B (CTxB) (Merritt et al., 1994) and we identified positive staining for GM1 with fluorophore-conjugated CTxB in the dorsal root and dorsal column white matter tracts, but also co-localising with markers of LacZ-affected nociceptor terminals from *Kcna6^lacZ/lacZ^* and Nav1.8^Cre/+^;ROSA26^*lacZ*/+^ mice in the superficial dorsal horn – including Cgrp and β-galactosidase (Fig. 14). Other co-localising ganglioside species were identified via monoclonal antibody staining against GD2 itself or GD3-based derivatives GT1b/GQ1b (Boffey et al., 2005; Clark et al., 2017) (Fig. 15).

### Mice lacking Kcna6 exhibit prolonged mechanical and thermal hypersensitivity after peripheral nerve injury

**Figure 16:**
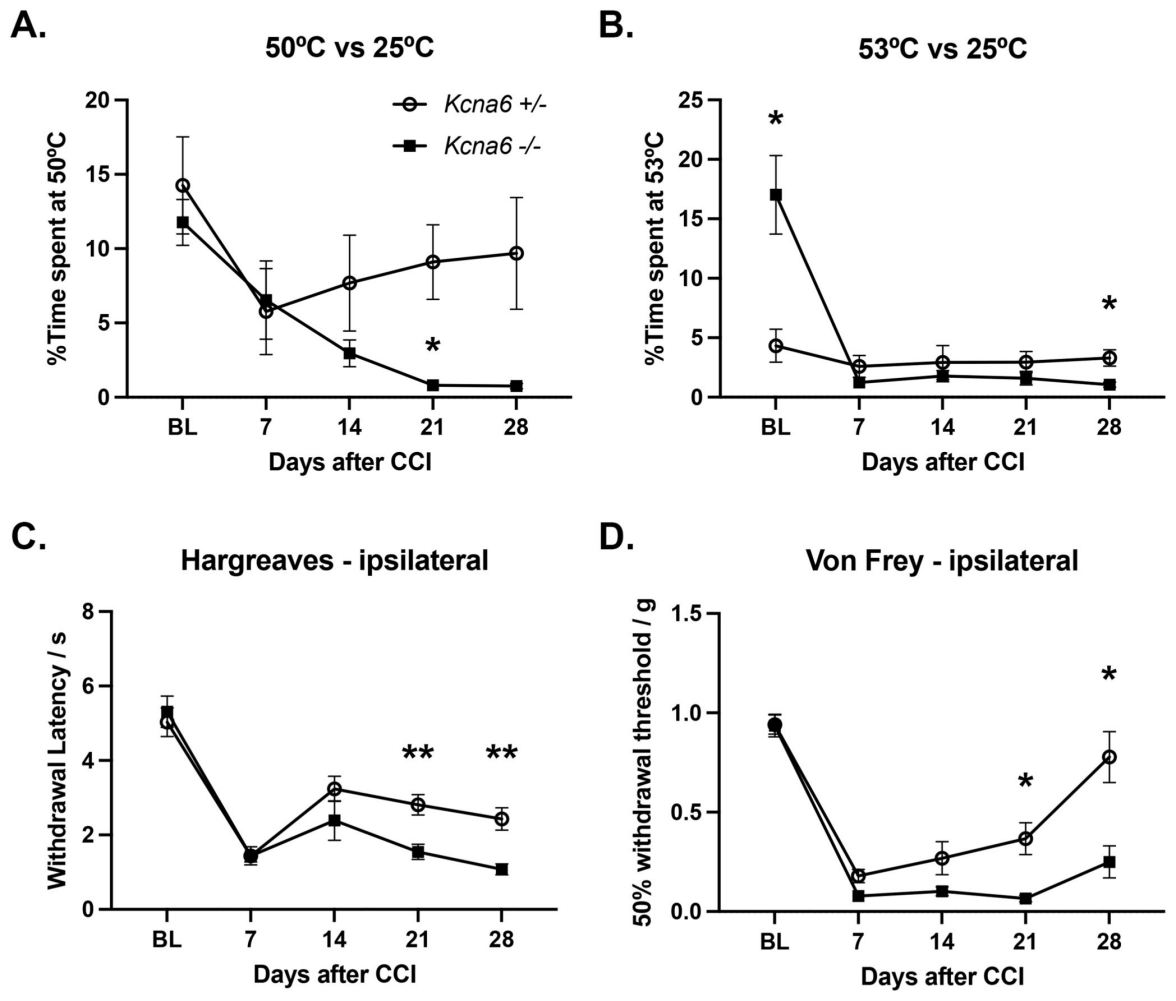
Mice lacking *Kcna6* suffer persistent mechanical and thermal hypersensitivity after chronic constriction injury of the sciatic nerve **(A, B)** Time spent at 50°C vs 25°C **(A)** or 53°C vs 25°C **(B)** was recorded over 2 minutes to assess thermal place preference before and after peripheral nerve injury in *Kcna6* ^+/-^ and *Kcna6* ^-/-^ mice. **(C)** The Hargreaves assay was used to assess thermal sensitivity thresholds in the hind paw ipsilateral to nerve injury. **(D)** Stimulation of the ipsilateral hind paw by Von Frey hairs was used to assess mechanical sensitivity thresholds. */** = p<0.05/0.01 *Kcna6* ^-/-^ vs *Kcna6* ^+/-^ by Two-Way Repeated Measures ANOVA with Geisser-Greenhouse correction for sphericity and Šidák post-hoc multiple comparison test. n = 10 *Kcna6* ^+/-^, 9 *Kcna6* ^-/-^ (age- and sex-matched). Data represented as mean ± SEM.

Upregulation of *Kcna6* in painful rat and human neuromas has implicated this subunit in a compensatory response to nerve injury (Calvo et al., 2016). Furthermore, local α-DTx mediated blockade of *Kcna6*-containing channels in the context of neuroma produces a persistent mechanical hypersensitivity (Calvo et al., 2016), revealing their role in setting neuropathic mechanosensory thresholds. We investigated whether this subunit contributes to pain sensation in a similar fashion in mice, using a chronic constriction injury (CCI) model in *Kcna6* ^em1(IMPC)J^ mice lacking *Kcna6* but not expressing *lacZ*. At baseline, as per Fig. 10, *Kcna6* knock-outs were again hyposensitive to 53°C but not 50°C noxious heat in a thermal place preference (TPP) assay versus 25°C (Fig. 16A,B), and displayed no difference in sensitivity to Hargreaves or Von Frey assays compared to littermate controls retaining a functional *Kcna6* allele (Fig. 16A,B). Behavioural outcomes were followed for 28 days post-injury. Across 50°C TPP, Hargreaves, and Von Frey assays, both cohorts developed thermal and mechanical hypersensitivity by 7 days post-injury (Fig. 16A,C,D). With follow-up across all of these assays, mice completely lacking *Kcna6* remained persistently hypersensitive in comparison to littermate controls, which displayed a degree of recovery from the initial day 7 hypersensitivity (Fig. 16A,C,D). Only homozygous *Kcna6*-null mice exhibited an observable injury-mediated hypersensitivity on 53°C vs. 25°C TPP due to their baseline hyposensitivity; however, in the post-injury period there were discernible differences between *Kcna6* ^-/-^ and *Kcna6* ^+/-^ mice at day 28 whereby *Kcna6*-null mice were hypersensitive compared to littermate controls (Fig. 16B).

## Discussion

### Neurotoxicity of exogenous LacZ expression

Through investigation of several transgenic mouse strains, a major finding was that exogenous *lacZ* cassettes expressed in primary afferent nociceptors cause pathological accumulation of lipid species within central presynaptic arborisations. These lipid-rich ‘organelles’ resemble endo-/lyso-/autophagosomal structures involved in turnover of plasma membrane gangliosides (Fig. 8, Fig. 12). Loss of function mutations affecting GM1-β-galactosidase or the group of enzymes that catabolise GM2 (β-hexosaminidases) result in fatal neurodegenerative ganglioside storage disorders (Breiden and Sandhoff, 2019), with accumulation of the substrates GM1 and GM2, respectively, in neuronal lysosomes.

GM1- and GM2-gangliosidoses feature “ballooned” axons (Breiden and Sandhoff, 2019), visible by Golgi-staining in feline models (Purpura and Baker, 1977; Walkley et al., 1981) but also present in post-mortem brain tissue from human patients (Sandhoff and Harzer, 2013). Ultrastructurally, these ‘meganeurites’ contain electron-dense membranous whorls and lysosome-like structures (Purpura and Baker 1977; Purpura, Pappas, and Baker 1978; Sango et al. 1995; Walkley, Wurzelmann, and Purpura 1981; Walkley, Baker, and Rattazzi 1990) remarkably similar to swollen terminals of *Kcna6^lacZ/lacZ^* and Nav1.8^Cre/+^;ROSA26^*lacZ*/+^ afferents (Fig. 8C-J, Fig. 12E,F). Similar vacuolated “inclusion bodies” were identified via TEM in DRG neurons from α-galactosidase knock-out mice in a Fabry disease model (Miller et al., 2018).

Endogenous β-galactosidase is undetectable in wild-type primary afferents by immunohistochemistry (Fig. 2), or RNA sequencing (Zeisel et al., 2018). We argue that exogenous LacZ/β-galactosidase overrides endogenous ganglioside metabolism, resulting in aberrant accumulation of several ganglioside species identified via immunostaining (Fig. 14, Fig. 15). Within the DRG, GM1-gangliosides are mostly associated with large myelinated primary afferent neurons (Robertson and Grant, 1989), and GM1 staining is almost absent from the superficial dorsal horn in mice – although GM1 upstream precursors GD1a, GD1b, and GT1b are abundant (Vajn et al., 2013). We clearly labelled GM1, GD2 and GD3-derived gangliosides in LacZ-affected terminals, suggesting endogenous ganglioside metabolism was being diverted through abnormal biochemical pathways (Breiden and Sandhoff, 2019).

Endogenous GM1-β-galactosidase expression accumulates over time and is a marker of cellular senescence (Dimri et al., 1995; Lee et al., 2006; Geng et al., 2010). GM2-gangliosidoses, including Tay-Sachs disease and Sandhoff disease, have an early onset, although variants with delayed onset and reduced severity correlate with improved catabolic GM2 clearance (Leinekugel et al., 1992; Sandhoff and Harzer, 2013; Breiden and Sandhoff, 2019). LacZ-induced ganglioside accumulation may therefore accelerate cellular ageing processes, consistent with slower pathological progression in *Kcna6^lacZ^* ^/+^ heterozygotes (Fig. 5) and Nav1.8^Cre/+^;ROSA26^*lacZ*/+^ mice (Fig. 11B,C), which only expressed one *lacZ* allele. Another feature of GM2 gangliosidosis, demyelination, involves microglia (Ogawa et al., 2018), which were also detected adjacent to swollen *Kcna6^lacZ^* profiles (Fig. 4C).

Although LacZ was detectable in *Kcna6^lacZ/lacZ^* distal nerve terminals innervating glabrous skin (Fig. 6), structural abnormalities were only observed in central afferent axons, mostly once they had penetrated the spinal cord but occasionally in the dorsal root of severely affected, aged animals. This is curious and accentuates the polarised structure and function of sensory neurons. It remains unclear why presynaptic axons were affected specifically, but neurotransmitter release is dependent on effective lysosomal and ganglioside function (Hirai et al., 2005), impaired in acquired autoimmune diseases such Guillain-Barré syndrome (Buchwald et al., 2007; Plomp and Willison, 2009). Gangliosides such as GM1 can co-localise with presynaptic proteins in lipid rafts (Taverna et al., 2004), known to regulate exocytosis (Salaün et al., 2004). Age-related presynaptic accumulation of GM1 also initiates degenerative change via amyloid deposition (Yamamoto et al., 2008).

We noted a predominant effect on nociceptive versus non-nociceptive afferent terminals in *Kcna6^lacZ/lacZ^* mice which likely reflects the higher expression of Kv1.6 (and hence LacZ) in nociceptors (Fig. 1, Fig. 2). Peptidergic and non-peptidergic nociceptors form morphologically distinct specialised central boutons (Ribeiro‐Da‐Silva and Coimbra, 1982; West et al., 2015; Gutierrez-Mecinas et al., 2016; Larsson and Broman, 2019). The propensity for degeneration of IB4^+^ more than Cgrp^+^ terminals is interesting; it suggests these subpopulations may express distinct species of gangliosides or rely on differential ganglioside metabolism to maintain presynaptic bouton integrity. IB4-binding terminals retract following peripheral nerve injury (Tajti et al., 1988; Molander et al., 1996; Shehab et al., 2004), whereas both IB4^+^ and Cgrp^+^ terminals retract following systemic administration of HIV therapeutic stavudine (Huang et al., 2013). These findings might probe further investigation into the role of ganglioside metabolism in maintaining primary afferent synaptic structures, especially in the context of peripheral neuropathy.

Our focus has been on *lacZ* expression in DRG neurons; one previous study has investigated expression in cortical neurons and raised concerns regarding the use of LacZ reporters (Reichel et al., 2016). The authors found that *lacZ* expression in cortical glutamatergic neurons caused severe morphological and behavioural impairments such as decreased hippocampal volume, reduced dendritic branching in hippocampal neurons, and deficits in hippocampus-dependent memory. They did not report abnormal lipid accumulations in axon terminals, although it is possible that these were not specifically sought. Our combined findings from *lacZ* expression in central and peripheral neurons mean that anatomical, electrophysiological and behavioural data from transgenic mice expressing *lacZ* require interpretation with extreme caution. We advocate using alternative reporters such as GFP or YFP, and note we and others have not observed any abnormalities in primary afferent terminals of *Advillin*-EGFP mice (Sikandar et al., 2017; Hunter et al., 2018). This is particularly relevant as a significant proportion of the lines produced in high throughput phenotypic screens such as the International Mouse Phenotyping Consortium use LacZ as a reporter (Skarnes et al., 2011; Meehan et al., 2017).

### The role of Kv1.6 in thermosensation

This study also revealed a role for Kv1.6 in regulating thermal sensitivity. The unexpected neurodegenerative phenotype of LacZ-expressing animals was considered likely to underly the differences in severity of thermal behavioural phenotypes between *Kcna6^lacZ/lacZ^* and *Kcna6* ^-/-^ mice, especially since Gb3 ganglioside accumulation in Fabry disease models was shown to interfere with Trpv1 signalling and heat sensation (Hofmann et al., 2018). The intricate mechanisms through which gangliosides modulate pain sensation specifically, including regulation of ion channels and receptors, have been reviewed recently (Sántha et al., 2020). The common feature between the two strains was hyposensitivity to noxious heat. At present, it is unclear through what mechanism this hyposensitivity manifests.

Most known functions of Kv1 channels would instead predict a hypersensitive phenotype upon removal of Kv1.6. Its upregulation following peripheral nerve injury in wild-type animals reduces spontaneous electrical discharge in injured nerve fibres and behavioural hypersensitivity, while pharmacological blockade of Kv1.6 in this context maintains spontaneous discharge and persistent mechanical hypersensitivity (Calvo et al., 2016). Blockade of Kv1 currents in non-peptidergic nociceptors isolated from Mrgprd-GFP mice (where Kv1.6 is the major Kv1 subunit) also increased their maximal action potential firing rate substantially (Zheng et al., 2019). The channel’s acute effect on membrane excitability thus limits excitability, in line with canonical Kv1 channel properties in neurons (Browne et al., 1994; Smart et al., 1998; Glazebrook et al., 2002; Chi and Nicol, 2007; Robbins and Tempel, 2012; Hao et al., 2013; Dawes et al., 2018). We had therefore been expecting if anything thermal hypersensitivity.

It is possible that these paradoxical effects relate to subunit stoichiometry of heteromeric Kv1 channels, which can significantly influence activation thresholds. Kv1.2 subunits promote a more depolarised activation potential than Kv1.1 (Akhtar et al., 2002), and Kv1.6 a still more depolarised activation potential (Grupe et al., 1990) (V_0.5max_ for homomeric channels: Kv1.1 -33mV, Kv1.2 -26mV, Kv1.6 -17mV). Knock-out of Kv1.6 in sensory neurons could result in over-representation of Kv1.1/1.2 subunits and reduced neuronal excitability. It is noted that *Kcna6* is expressed throughout the mouse nervous system (Zeisel et al., 2018) and in two global Kv1.6 knock-out strategies there are myriad explanations for hyposensitive behavioural traits, requiring further interrogation.

### Kcna6 promotes recovery from nerve injury

Intriguingly, in the context of neuropathic chronic constriction injury *Kcna6*-null animals behave in a more canonical fashion, displaying more severe and prolonged hypersensitivity to thermal and mechanical stimuli, while littermate controls with functioning Kv1.6 gradually recover. We were able to observe the hyposensitive phenotype to acute noxious thermal stimuli reported in both *Kcna6* knock-out strains (Fig. 3, Fig. 10) and the persistent post-neuropathic hypersensitive phenotypes predicted by (Calvo et al., 2016), in the same cohort of animals (Fig. 16). We believe this reconciles the two contrasting datasets and provides robust evidence that both phenomena are genuine consequences of Kv1.6 loss of function.

## Significance Statement

In recent decades the expansion of technologies to experimentally manipulate the rodent genome has contributed significantly to the field of neuroscience. While introduction of enzymatic or fluorescent reporter proteins to label neuronal populations is now commonplace, often potential toxicity effects are not fully considered. We show a role of Kv1.6 in acute and neuropathic pain states through analysis of two mouse models lacking Kv1.6 potassium channels, one with additional expression of LacZ and one without. We show that LacZ reporter enzymes induce unintended defects in sensory neurons, with an impact on behavioural data outcomes. To summarise we highlight the importance of: Kv1.6 in recovery of normal sensory function following nerve injury, and careful interpretation of data from LacZ reporter models.

## Summary

We have identified a role for Kv1.6 subunits in acute thermal nociception and the recovery response to nerve injury. We also present evidence that transgenic insertion of exogenous *lacZ*-containing cassettes can have pathological downstream consequences for neuronal structure and function. LacZ induced accumulation of gangliosides in presynaptic primary nociceptor terminals in the dorsal horn. This anatomical defect exacerbated the underlying behavioural phenotype of Kv1.6 knock-out mice, demonstrated by comparison between *Kcna6^lacZ^* and CRISPR-mediated *Kcna6* ^-/-^ mice. This confirms that exogenous *lacZ* cassettes can be markedly neurotoxic. We recommend caution when interpreting past and future results from transgenic mice expressing exogenous LacZ. We also illustrate a role for Kv1.6 in the recovery of normal sensory function following nerve injury, thus enhancing Kv1.6 function could be a therapeutic target in neuropathic pain.
